# *Toxoplasma gondii* infection inhibits invasion and migration of human extravillous trophoblasts through dysregulation of FOXO1- and FOXO3a-dependent and -independent mechanisms

**DOI:** 10.3389/fcimb.2025.1651142

**Published:** 2025-12-10

**Authors:** Aurore Lebourg, Louis-Philippe Leroux, Visnu Chaparro, Sophie Chagneau, Cathy Vaillancourt, Maritza Jaramillo

**Affiliations:** 1 Institut National de la Recherche Scientifique (INRS)-Centre Armand-Frappier Santé Biotechnologie (CAFSB), Laval, QC, Canada; 2 Centre intégré universitaire de santé et de services sociaux-Nord-de-l’île-de-Montréal, Montréal, QC, Canada

**Keywords:** *Toxoplasma gondii*, extravillous trophoblasts, invasion, migration, FOXO3a, FOXO1, AKT

## Abstract

The protozoan parasite *Toxoplasma gondii* causes severe pathologies in the infected fetus following vertical transmission during pregnancy. Primary *T. gondii* infection increases the risk of miscarriage during the first trimester of gestation; however, the cellular and molecular mechanisms are not fully understood. Extravillous trophoblasts (EVTs) are fetal cells that migrate and invade the maternal decidua to allow placenta formation and embryo implantation. The transcription factors Forkhead box O3a (FOXO3a) and FOXO1 play a central role in the regulation of EVT migration and invasion. Hence, impairment of EVT functions is associated with FOXO3a/FOXO1 dysregulation and poor pregnancy outcomes. Interestingly, *T. gondii*-driven inactivation of FOXO3a and FOXO1 was reported in fibroblasts and macrophages. Using a combination of cell imaging, reverse genetics and biochemical approaches in the human trophoblast cell line HTR-8/SVneo, herein we provide evidence that infection with type I RH *T. gondii* strain inhibits invasiveness and migratory activities in EVTs by repressing FOXO3a-and FOXO1-dependent and independent gene expression. Indeed, either *T. gondii* infection or single knockdown of FOXO3a and FOXO1 reduced invasion and migration properties in EVTs. Selective chemical blockade of parasite motility and host cell entry indicates that active infection is indispensable for reduced EVT migration but is only partially required for *T. gondii*-driven repression of EVT invasiveness. Mechanistically, *T. gondii* infection led to AKT-sensitive phosphorylation and nuclear exclusion of FOXO3a and FOXO1 in EVTs. An RT-qPCR-based screening identified a subset of invasion-and migration-associated genes downregulated in *T. gondii*-infected EVTs (MMP2, MMP3, MMP14, TIMP2, MUC1, and ITGB3). Transcription of genes encoding MMP3 and integrin β3 decreased in FOXO3a KD and FOXO1 KD EVT cell lines, respectively. These data along with pharmacological inhibition of AKT in infected cells provide evidence that *T. gondii* downregulates MMP3 and ITGB3 gene expression in EVTs in an AKT-FOXO3a/FOXO1-dependent fashion. In all, we have uncovered a novel regulatory mechanism involved in the repression of EVT migration and invasion properties during *T. gondii* infection. Further investigation using *in vivo* and *ex vivo* models of placental infection is required to determine whether *T. gondii*-driven dysregulation of EVT functions contributes to pregnancy complications associated with congenital toxoplasmosis.

## Introduction

The obligate intracellular parasite *Toxoplasma gondii* (*T. gondii*) belongs to a group of human pathogens collectively known as TORCH (i.e., *T. gondii*, rubella virus, cytomegalovirus, and herpes virus), which have the capacity to cross the placental barrier and cause severe congenital pathologies associated with primary infection acquired during pregnancy (Ostrander and Bale, 2019). The consequences of congenital toxoplasmosis range from early-trimester miscarriage to late-trimester neurological and ocular fetal and perinatal defects (e.g., retinochoroiditis, hydrocephalus, mental retardation, etc.) (Borges et al., 2019). It is estimated that the global annual incidence of congenital toxoplasmosis is ~190,000 cases, accounting for 1.2 million disability-adjusted life years (DALYS) per year (Rostami et al., 2019). Unfortunately, there are no effective vaccines approved for human use and current treatments are teratogenic (Zhang et al., 2022b; Dunay et al., 2018). Hence, there is an urgent need to better understand the molecular mechanisms that regulate interactions between *T. gondii* and the host within the placental microenvironment to identify safer and more efficient treatments.

At the maternal-fetal interface, *T. gondii* infects extravillous trophoblasts (EVTs) (Fallahi et al., 2018), a fetal cell population that anchors the placenta to the uterine implantation site (i.e., decidua) and is therefore essential for normal placentation and embryo development (Ashton et al., 2005; He et al., 2017; Erlebacher, 2013). In addition to EVTs, *T. gondii* is also able to infect other subpopulations of trophoblasts (i.e., villous cytotrophoblasts and syncytiotrophoblast); however, EVTs appear to be particularly susceptible to the infection (Robbins et al., 2012; Almeida et al., 2019). Accumulating evidence indicates that *T. gondii* modulates host cell signaling pathways and biological functions to establish a safe replicative niche within EVTs (Milian et al., 2019; de Souza et al., 2021; Almeida et al., 2019; Guirelli et al., 2015). For instance, it was shown that *T. gondii* intracellular proliferation relies on the sequential expression of macrophage inhibitory factor (MIF) and ERK1/2-dependent synthesis of COX-2 in the human trophoblast cell line HTR-8/SVneo (Milian et al., 2019). Consistent with this, it was described that *T. gondii* triggers a COX-2-PGE_2_-mediated anti-inflammatory cytokine response that promotes parasite growth in human EVTs (de Souza et al., 2021). In parallel, enhanced transcription of the gene encoding the transferrin receptor (*TFRC*) was reported in *T. gondii*-infected HTR-8/SVneo cells (Almeida et al., 2019), suggesting that the parasite hijacks iron metabolism in EVTs. Conversely, EVTs deploy defense mechanisms to control *T. gondii* infection, including reactive oxygen species (ROS) production, inflammasome activation, and pyroptosis (Quan et al., 2023).

The ability of EVTs to migrate and invade the maternal decidua is required for (i) reshaping decidual spiral arteries to ensure sufficient blood flow and oxygen concentration (Ashton et al., 2005), (ii) allowing nutrient exchange between mother and fetus, and (iii) promoting immune cell interactions necessary for an immunotolerant environment (He et al., 2017; Erlebacher, 2013). Dysregulation of these biological processes is known to contribute to pregnancy-related pathologies, such as preeclampsia or placenta accreta spectrum (Kudo and Sugimoto, 2024). EVT migration and invasion properties require the activity of diverse transcription factors (e.g., FOXO3a, FOXO1, SNAIL, STAT3, β-catenin, etc.) (Chen et al., 2022, 2021, 2018; Fitzgerald et al., 2008; Han et al., 2019; Li et al., 2015), adhesion molecules (e.g., ICAM, MUC-1, integrin β3, integrin β1, etc.) (Thirkill et al., 2007; Shyu et al., 2011; Chen et al., 2018), and metalloproteinases (MMPs) that degrade the extracellular matrix (e.g., MMP-2, MMP-3, MMP-9, pappalysin-1, etc.) (Zhang et al., 2020a; Chen et al., 2023; Fitzgerald et al., 2008; Li et al., 2015; Suman et al., 2013; Demir-Weusten et al., 2007; Onogi et al., 2011).

The transcription factors Forkhead box O3a (FOXO3a) and FOXO1 play crucial roles in decidualization and embryo implantation. In addition, FOXO3a and FOXO1 are central regulators of EVT functions, including invasion, migration, and related gene expression programs (e.g., *ITGB3*, *ICAM1*, *MMP9*, etc.) (Chen et al., 2022, 2021; Long et al., 2019; Vasquez et al., 2018; Kajihara et al., 2006). Hence, dysregulation of FOXO3a and FOXO1 has been associated with poor pregnancy outcomes, such as preeclampsia and gestational diabetes mellitus (Zhang et al., 2020b; Chen et al., 2021; Zhang et al., 2022a; Chen et al., 2018; Ding et al., 2023; Akkaya Firat et al., 2025). In contrast to FOXO3a and FOXO1, activation of the TGF-β1-SMAD2/3 signaling pathway has been shown to inhibit HTR-8/SVneo cell invasiveness by preventing transcription of invasion-related genes (i.e., connective tissue growth factor and kisspeptin) (Fang et al., 2022; Cheng et al., 2017). Similarly, the transcription factor GATA2 was described as a repressor of invasion and associated genes in human EVTs (Ogoyama et al., 2021).

Interestingly, *T. gondii* infection has been reported to downregulate adhesion molecule expression (e.g., integrin β1) in human monocytes (Cook et al., 2018) and genes encoding MMPs (e.g., *MMP1*, *MMP3*, etc.) in human decidual macrophages (Fu et al., 2024). Moreover, inactivation of FOXO3a- and FOXO1-dependent host autophagic responses were described in *T. gondii-*infected human fibroblasts (Diez et al., 2023) and murine macrophages (Lee et al., 2022), respectively. Conversely, increased SMAD2 phosphorylation and TGF-β1 secretion were detected in *T. gondii*-infected macrophages (Damasceno-Sá et al., 2021) and in human trophoblast cell line HTR-8/SVneo (de Souza et al., 2021). In all, these studies indicate that *T. gondii* alters migration- and invasion-related signaling pathways, transcription factors, and gene expression programs in different host cell types. However, the ability of *T. gondii* to regulate EVT migratory and invasive properties and its molecular basis have yet to be investigated. Here, we show that *T. gondii* infection leads to reduced migration and invasiveness of HTR-8/SVneo, an EVT model. Mechanistically, these events involve parasite-driven AKT-sensitive nuclear exclusion of FOXO3a and FOXO1 and repression of FOXO3a/FOXO1-dependent and -independent transcription of invasion- and migration-related genes.

## Materials and methods

### Reagents

Culture media and supplements were purchased from Wisent and Gibco; MK-2206 was obtained from Cayman Chemical; SB431542 was purchased from Selleck Chemicals; mycalolide B was obtained from Fujifilm; cOmplete EDTA-free protease inhibitor and PhosSTOP phosphatase inhibitor tablets were obtained from Roche; DAPI was purchased from Invitrogen; human TGF-β1 recombinant protein was obtained from Cell Signaling Technologies; rat tail collagen type-I was purchased from Gibco.

### Parasite maintenance and harvest

*T. gondii* RH and ME49 strains were maintained by serial passage in Vero cells, as previously described (Diez et al., 2023). Cultures were grown in DMEM with 5% heat-inactivated FBS, 2 mM L-glutamate, 1 mM sodium pyruvate, 100 U/mL penicillin, and 100 µg/mL streptomycin, and incubated at 37 °C, 5% CO_2_. For experimental infections, freshly egressed tachyzoites were harvested, pelleted by centrifugation (1,300 × *g*, 7 min, 4 °C), resuspended in ice-cold PBS (pH 7.2-7.4), and passed through a syringe fitted with a 27 G needle. After removing large debris and intact host cells via low-speed centrifugation (200 × *g*, 3 min, 4 °C), the supernatant containing parasites was filtered through a 3-µm polycarbonate filter (Millipore). Tachyzoites were washed twice in PBS and finally resuspended in the appropriate culture medium, according to the experiment.

### Soluble *T. gondii* antigens and heat-killed parasites

STAgs and HK parasites were prepared from freshly egressed tachyzoites, as previously described (Diez et al., 2023). For STAgs, parasites were resuspended in ice-cold PBS, subjected to three 5-min cycles of freezing/thawing using liquid nitrogen and a 37 °C water bath, and then sonicated on ice for 5 min (1 s on/off pulses, 30% duty cycle) using a Sonic Dismembrator FB505 (ThermoFisher). After centrifugation (21,000 × *g*, 15 min, 4 °C), soluble material containing STAg was collected and used for downstream experiments. HK parasites were prepared by incubation at 56 °C for 10 min. Parasites were then pelleted by centrifugation (1,300 × *g*, 7 min, RT) and resuspended in the appropriate culture medium according to the experiment.

### Parasite treatment with mycalolide B

*T. gondii* cultures were treated with mycalolide B prior to infection, as described (Rosowski et al., 2014). Briefly, freshly egressed tachyzoites were incubated with 3 μM mycalolide B or vehicle (i.e., DMSO) for 15 min at 37 °C and washed twice in warm PBS (pH 7.2-7.4). Parasites were then pelleted by centrifugation (1,300 × *g*, 7 min, RT) and resuspended in cell culture media for downstream experiments.

### Infection and treatment of HTR-8/SVneo cells

HTR-8/SVneo cells (ATCC # CRL-3271) were plated one day before infection in DMEM supplemented with 10% FBS, 2 mM L-glutamate, 1 mM sodium pyruvate, 100 U/mL penicillin, and 100 µg/mL streptomycin at 37 °C, 5% CO_2_. Cultures were serum-starved for 1 h, then inoculated with live or HK parasites (multiplicity of infection [MOI] of 1:1, 2:1, 5:1), treated with 50 μg/mL STAgs, or left uninfected in fresh medium without FBS. Any remaining extracellular parasites were rinsed away with warm PBS (pH 7.2-7.4) 1 h following inoculation, fresh medium without FBS was added with inhibitors or vehicle when applicable, and cells were incubated until experiment completion.

### Viability assays

Viability of HTR-8/SVneo cells was determined by the resazurin assay as described (Diez et al., 2023). Briefly, cells were treated with increasing concentrations of MK-2206 (0.0625–32 µM), SB431542 (0.195-200 µM) or an equivalent volume of DMSO (vehicle) for 24 h at 37 °C, 5% CO_2_. The medium was removed and replaced with fresh culture medium supplemented with 0.025% resazurin. Cultures were incubated for 4 h in the presence of the inhibitors or DMSO for 4 h at 37 °C, 5% CO_2_. Optical density was measured using a Multiskan GO (ThermoFisher) at 600 and 570 nm. Absorbance at 600 nm was subtracted from readings at 570 nm. Experiments were performed in three biological replicates (*n* = 3); each sample was analyzed in a technical triplicate.

### Invasion assays

Trophoblast cell invasiveness was monitored by a collagen-based matrix invasion assays as described (Chen et al., 2021, 2022). Briefly, the upper chamber of 8-μm pore size membrane inserts (Corning) was coated with 5 μg/cm² rat tail collagen-I (Gibco ThermoFisher Scientific) diluted in sterile PBS and incubated at room temperature at least 4 h prior to invasion assays. HTR-8/SVneo cells were infected or treated with different stimuli for 4 h and were collected by centrifugation (200 × *g*, 7 min, 4 °C). Approximately 7.5 × 10^4^ cells were resuspended in serum-free DMEM and were seeded in the insert upper chamber. DMEM containing 10% FBS was added to the insert lower chamber to induce cell invasion. Following a 16 h incubation period, non-invasive cells that remained in the upper chamber were removed with a cotton swab. Cells that had migrated through the matrigel towards the bottom chamber were gently rinsed with PBS. Inserts membranes were then fixed in 2% paraformaldehyde (PFA) in PBS for 10 min at RT and stained with Hema 3 staining (ThermoFisher Scientific). Cell invasion was visualized by light microscopy with a 20x objective of a Nikon Eclipse microscope. Images were acquired from four different fields of view per treatment with the NIS-element software and analyzed with ImageJ (NIH, USA). Briefly, the area of invasion was analyzed by applying a manual threshold to detect the Hema 3 staining. Cell viability was determined prior to cell seeding in the matrigel and following invasion assays by cell counting using trypan blue staining.

### Cell migration assays

*In vitro* wound-healing assays were carried out to measure trophoblast cell migration as described (Chen et al., 2021, 2022). Briefly, HTR-8/SVneo cells were seeded in 6-well tissue culture plates at a starting density of 1 × 10^6^ cells/well and were cultured overnight to reach 90% confluence. Cells were infected or treated with different stimuli for 1 h and monolayers were then scratched with a 1,000 μL pipette tip to create a cross-shaped wound (Dallagi et al., 2015). Cultures were washed with PBS at RT and fresh serum-free DMEM was added. Images were acquired at 0 and 16 h post-scratch wound by light microscopy with a 4x objective of a Nikon Eclipse microscope using the NIS-element software. To measure the area of wound closure, images were taken from the same area and cells bordering the wounds were traced using ImageJ software “wound-healing assay size tool”. The area of migration was calculated from the equation ((Wi-Wz)/Wi) × 100; where Wi is the area of wound at t = 0 h and Wz is the area of wound closure after 16 h. Percentage of migration was normalized to uninfected control cultures or scrambled shRNA-transduced cell lines according to the experiment. In parallel, cells were collected at 0 and 16 post-scratch wound and stained with trypan blue to count live cells at each time point and calculate the proliferation ratio.

### Western blot analysis

Following infection and other treatments, cells were lysed directly in RIPA lysis buffer (25 mM Tris (pH 7.6), 150 mM NaCl, 1% Triton-X 100, 0.5% sodium deoxycholate, 0.1% SDS) supplemented with cOmplete EDTA-free protease inhibitor, PhosSTOP phosphatase inhibitor tablets and 10 mM 1,10-phenanthroline monohydrate (Sigma-Aldrich). Lysates were stored at -80 °C or diluted with a solution of Laemmli buffer, heated at 95 °C for 5 min and processed immediately for SDS-PAGE. Resolved proteins were transferred onto PVDF membranes. Membranes were blocked for 1 h at RT in TBS 0.1% Tween-20 (TBS-T), 5% skim milk, then probed with the following primary antibodies: anti-phospho-AKT (S473) (clone D9E, #4060), anti-phospho-AKT (T308) (clone C31E5E, #2965), anti-AKT (clone C67E7, #4691), anti-phospho-FOXO3a (S253) (clone D18H8, #13129), anti-phospho-FOXO3a/FOXO1 (T32/T24) (polyclonal, #9464), anti-FOXO1 (clone C29H4, #2880), anti-phospho-FOXO3a (S253) (clone D18H8, #13129), anti-FOXO3a (clone 75D8, #2497), anti-phospho-SMAD2 (S465/S467) (clone E8F3R, #18338), anti-SMAD2 (clone D43B4) #5339, anti-Lamin B1 (clone D4Q4Z, #12586), anti-α-tubulin (clone DM1A, #3873), anti-GATA2 (clone 79802S, #79802S), and β-actin (clone 8H10D10, #3700) were obtained from Cell Signaling Technologies; anti-*T. gondii* profilin-like protein (polyclonal, #AF3860) was purchased from R&D Systems. Membranes were then probed with the following horseradish peroxidase (HRP)-conjugated antibodies: goat anti-rabbit IgG (#A0545), goat anti-mouse IgG (#A4416) (Sigma-Aldrich) or rabbit anti-goat (R&D Systems). Subsequently, proteins were visualized using the Clarity ECL Western blotting substrate (Bio-Rad) and exposing the membranes to autoradiography film (Denville Scientific).

### Preparation of HTR-8/SVneo nuclear and cytoplasmic extracts

Following infection and other treatments, cells were gently scraped in ice-cold PBS 5 mM EDTA (supplemented with cOmplete EDTA-free protease inhibitor, PhosSTOP phosphatase inhibitor tablets, and 10 mM 1,10-phenanthroline [Sigma-Aldrich]). A fraction of the cell suspensions was kept aside to prepare Whole Cell Lysates (WCL); these samples were lysed and processed for western blotting as detailed above. Samples were centrifuged (500 × *g*, 5 min at 4 °C), and the pellets were resuspended in 300 μL of ice-cold Hypotonic Lysis Buffer (HBL) (10 mM HEPES, 10 mM KCl, 0.1 mM EDTA, 0.1 mM EGTA, 1 mM DTT, cOmplete EDTA-free protease inhibitor, PhosSTOP phosphatase inhibitor tablets, and 10 mM 1,10-phenanthroline). Samples were incubated for 15 min on ice with regular mixing, then 30 μL of 10% IGEPAL CA-630 was added to suspensions. After centrifugation (16,000 × *g*, 1 min,4 °C), supernatants (containing the cytoplasmic contents) were transferred to new tubes. Pellets were washed twice by adding 500 μL HBL (without IGEPAL CA-63) and centrifuged (16,000 × *g*, 1 min,4 °C). Pellets were then lysed in 60 μL of Nuclear Lysis Buffer (NLB) (20 mM HEPES, 400 mM NaCl, 1 mM EDTA, 1 mM EGTA, 1 mM DTT, 5% glycerol, cOmplete EDTA-free protease inhibitor, PhosSTOP phosphatase inhibitor tablets, and 10 mM 1,10-phenanthroline). After 15 min of incubation on ice with occasional vortexing, lysates were centrifuged, and supernatants (containing the nuclear material) were transferred to new tubes. Samples were next processed for western blot analyses.

### Immunofluorescence and confocal microscopy

HTR-8/SVneo cultures were seeded onto glass coverslips in 24-well plates overnight. At the indicated time points, cells were rinsed with PBS three times and then fixed with 3.7% PFA in PBS for 15 min at RT. Cells were permeabilized with 0.2% Triton X-100 (in PBS) for 5 min at RT for total FOXO1 and FOXO3a staining. Cells were permeabilized with 100% methanol for 20 min at -20 °C for phospho-SMAD2 (S465/467) staining. Samples were kept in blocking solution (5% skim milk, 1% BSA, 5% normal goat serum in PBS) for 1 h at RT and incubated for 2 h at RT with the following primary antibodies diluted in PBS with 1% BSA: anti-FOXO3a (clone 75D8, #2497), anti-FOXO1 (clone C29H4, #2880), and anti-phospho-SMAD2 (S465/S467) (clone E8F3R, #18338) were obtained from Cell Signaling Technologies. Samples were then incubated with the following fluorochrome-conjugated secondary antibody for 2 h at RT: donkey anti-rabbit IgG (H+L) Alexa Fluor 647 (#A-31573) was purchased from Invitrogen. Nuclei were stained with 300 nM DAPI (Invitrogen, #D21490) for 5 min at RT. Coverslips were mounted onto slides with Fluoromount G (#00-4958-02, Invitrogen). Samples were visualized with a 40x objective of a LSM780 Zeiss confocal microscope, image acquisition was carried out using ZEN software, and image processing was performed with ImageJ.

### Purification of RNA and quantitative RT-PCR

Total cellular RNA was extracted with QIAzol (Qiagen) according to the manufacturer’s specifications. RNA concentration and purity were assessed spectrophotometrically by using a NanoDrop One instrument (Thermo Fisher Scientific). Approximately 1 μg of RNA was reverse transcribed using LunaScript^®^ RT SuperMix Kit (New England Biolabs). Real-time quantitative PCR (RT-qPCR) was performed with Luna^®^ qPCR Master Mix (New England Biolabs) using a QuantStudio 3 Real-Time PCR System (Thermo Fisher Scientific). Relative quantification was calculated using the comparative Ct method (ΔΔCt) (Taylor et al., 2010) and relative expression was normalized to human *HPRT1*. Primers used for RT-qPCR were designed using NCBI Primer-BLAST (http://www.ncbi.nlm.nih.gov/tools/primer-blast/) and are listed in **Supplementary Table S1**.

### Lentivirus production and HTR-8/SVneo transduction

Lentiviruses were produced in HEK-293T cells using Lenti-Pac HIV Expression Packaging Kit, as per manufacturer’s guidelines (GeneCopoeia; #LT001). Lentivirus titers were measured using Lenti-Pac HIV qRT-PCR Titration Kit (GeneCopoeia; #LT005) according to the manufacturer’s protocol. To generate FOXO1 and FOXO3a KD cells, HTR-8/SVneo cells were transduced with the different lentivirus preparations for 3 days in the culture medium supplemented with 5 µg/mL polybrene (hexadimethrine bromide). Transduced cultures were selected for 6 days using 2 µg/mL puromycin to generate stable cell lines used immediately for downstream experiments. Plasmids and shRNA clones were purchased from GeneCopoeia (**Supplementary Table S2**).

### Statistical analysis

Data are presented as the mean (standard deviation) (SD) of the mean. Statistical significance was determined by using one-way or two-way ANOVA followed by a Tukey *post hoc* test or a two-tailed independent Student’s *t*-test; calculations were performed by using Prism 7 software package (GraphPad). Differences were considered significant when *P < 0.05, ** *P* < 0.01, *** *P* < 0.001, and **** *P* < 0.0001.

## Results

### *Toxoplasma gondii* inhibits invasion and migration properties, and related genes in human trophoblasts

**Figure 1:**
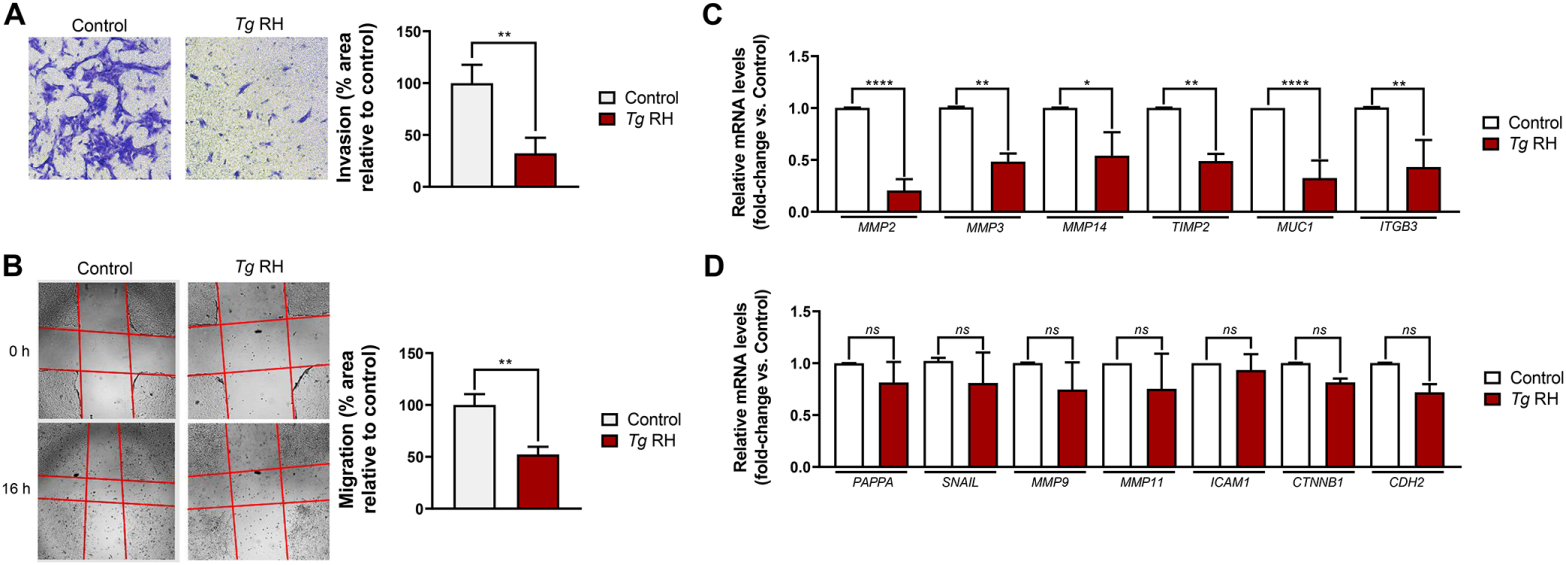
*T. gondii* downregulates invasion, migration, and associated genes in the human trophoblast cell line HTR-8/SVneo. Cells were incubated in serum-free media 1 h before infection with *T. gondii* RH strain (*Tg* RH) at MOI 5:1 or left uninfected (Control). **(A)** Collagen-based matrix invasion was monitored at 16 hours post-infection (h.p.i). Shown here are representative images of three independent experiments (left panel). Quantification of cell invasion using the ImageJ software (right panel). **(B)** Cell migration was assessed by wound-healing assays. Representative images of three independent experiments (left panel). Quantification of cell migration using ImageJ software (right panel). **(C, D)** Relative amounts of indicated invasion- and migration-related transcripts were quantified by RT-qPCR. Relative expression was normalized to *HPRT1*. **(A–D)** Values are presented as mean [SD] calculated from three independent experiments (n = 3). Statistical significance was determined using a two-tailed independent Student’s *t-*test **(A, B)** or a one-way ANOVA, followed by *post-hoc* Tukey’s test **(C, D)**, where ****P < 0.0001; **P < 0.01; *P < 0.5; *ns* not significant.

HTR-8/SVneo is an immortalized human EVT cell line widely used as a model system to study invasion and migration (Abbas et al., 2020), two essential processes for placenta implantation, development and healthy pregnancy (Knofler et al., 2019). Given that EVTs are preferentially infected by *T. gondii* in an *in vitro* placental model (Robbins et al., 2012), we set out to elucidate whether the parasite interferes with two of their main biological functions; namely, invasive and migratory capacities. To test this, HTR-8/SVneo cells were infected with RH *T. gondii* tachyzoites at multiplicity of infection (MOI) 5:1 and carried out collagen-based matrix invasion and “wound-healing” migration *in vitro* assays. A significant decrease in EVT invasion and migration was detected at 16 hours post-infection (h.p.i) (**Figures 1A, B**, respectively). These effects were not attributed to reduced cell viability nor to changes in proliferation rate, as no differences were observed between infected and uninfected control cells (**Supplementary Figures S1A, B**, respectively). HTR-8/SVneo infection with increasing parasite – host cell ratios revealed that *T. gondii*-driven downregulation of EVT invasion and migration is detectable at MOI 2:1 but is more pronounced at MOI 5:1 (**Supplementary Figure S2**).

Alteration of biological functions in *T. gondii*-infected cells at the maternal-fetal interface has been linked to dysregulation of host gene expression (Fu et al., 2024; Hoo et al., 2024). Hence, we postulated that *T. gondii* downregulates transcriptional programs associated with invasion and migration in human EVTs. Our RT-qPCR analyses revealed decreased expression of a subset of transcripts encoding proteins related to invasion and migration (i.e., *MMP2*, *MMP3*, *MMP14*, *TIMP2*, *MUC1*, and *ITGB3*) in *T. gondii*-infected HTR-8/SVneo cells (**Figure 1C**). However, our screening also identified invasion- and migration-related genes that were not significantly modulated upon infection (i.e., *PAPPA*, *SNAIL*, *MMP9*, *MMP11*, *ICAM1*, *CTNNB1*, and *CDH2*) (**Figure 1D**). These data provide evidence that repression of EVT invasion and migration by *T. gondii* correlates with dysregulation of specific gene expression programs.

**Figure 2:**
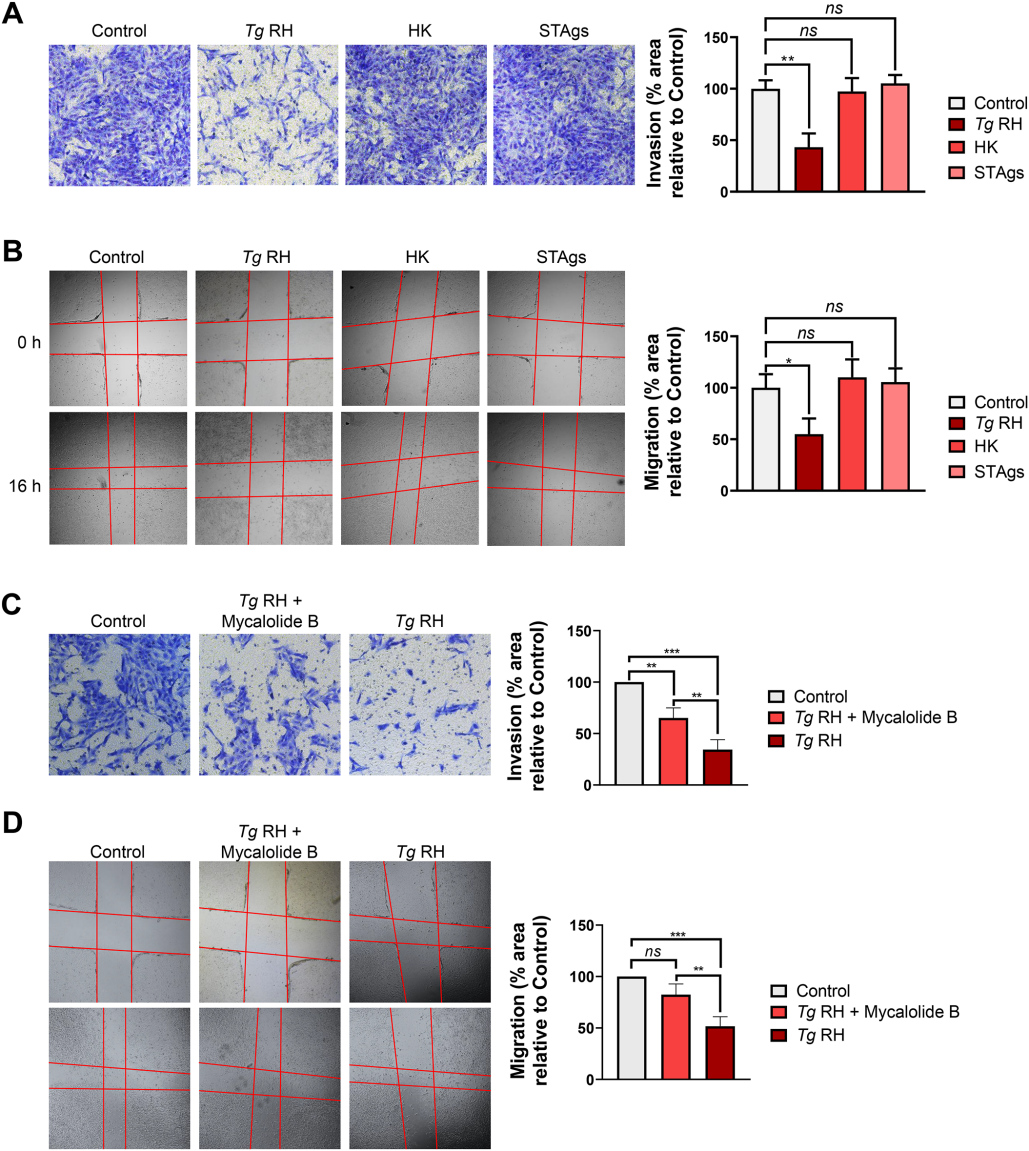
Live infection is indispensable for *T. gondii*-driven inhibition of migration but is partially required for reduced invasiveness in the human trophoblast cell line HTR-8/SVneo. Serum-starved HTR-8/SVneo cultures were inoculated with live or heat-killed (HK) *Tg* RH, treated with 50 μg/mL soluble *T. gondii* antigens (STAgs) **(A, B)**, incubated with DMSO- or mycalolide B-pretreated *Tg* RH tachyzoites **(C, D)** or left uninfected and untreated (Control) **(A–D)** for 16 h. **(A, C)** Cell invasiveness was monitored at 16 h.p.i./post-treatment. Shown here are representative images of three independent experiments (left panels). Cell invasion was quantified using the ImageJ software (right panels). **(B, D)** Cell migration was assessed at 16 h.p.i./post-treatment by wound-healing assays. Shown here are representative images of three independent experiments (left panels). Quantification of cell migration using ImageJ (right panels). **(A–D)** Values are presented as mean [SD] calculated from three independent experiments (n = 3). Statistical significance was determined using a one-way ANOVA, followed by *post-hoc* Tukey’s test, where ***P < 0.001; **P < 0.01; *P < 0.05; *ns*, not significant.

We next sought to determine whether live infection was required for the dysregulation of invasion and migration in EVTs. In contrast to infection by live tachyzoites, heat-killed (HK) parasites and soluble *T. gondii* antigens (STAgs) had no effect on the invasive and migration properties of HTR-8/SVneo cells (**Figures 2A, B**, respectively). To further investigate the requirement of live infection for *T. gondii*-driven modulation of EVT functions, we pretreated freshly isolated parasite cultures with mycalolide B, which prevents host cell entry without blocking rhoptry- and microneme-dependent protein secretion (Cirelli et al., 2014; Rosowski et al., 2014). We initially assessed the efficacy of mycalolide B treatment by performing RT-qPCR experiments. Consistent with reduced parasite internalization, we observed a marked decrease in *T. gondii TUB1A* mRNA abundance in HTR-8/SVneo cultures incubated with mycalolide B-pretreated parasites compared to cells infected with *T. gondii* tachyzoites pretreated with vehicle (i.e., DMSO) (**Supplementary Figure S3A**). We then tested the effect of mycalolide B treatment in EVT invasion and migration impairment by *T. gondii*. Incubation with mycalolide B-pretreated tachyzoites or infection with untreated parasite cultures led to a marked decrease in HTR-8/SVneo cell invasiveness (**Figure 2C**); however, the inhibitory effect was significantly attenuated upon mycalolide B pretreatment. In contrast to control *T. gondii* cultures, mycalolide B-pretreated parasites failed to inhibit HTR-8/SVneo cell migration (**Figure 2D**). Of note, no changes were detected in EVT viability and proliferation upon HK and STAg treatment (**Supplementary Figures S3B, C**, respectively) or incubation with mycalolide B-pretreated parasites (**Supplementary Figures S3D, E**, respectively). Taken together, this set of experiments indicates that reduced EVT invasion by *T. gondii* is partially dependent on host cell entry whereas parasite-driven impairment of EVT migration requires active live infection.

### Downregulation of invasion-related genes in *T. gondii*-infected human trophoblasts is SMAD2- and GATA2-independent

**Figure 3:**
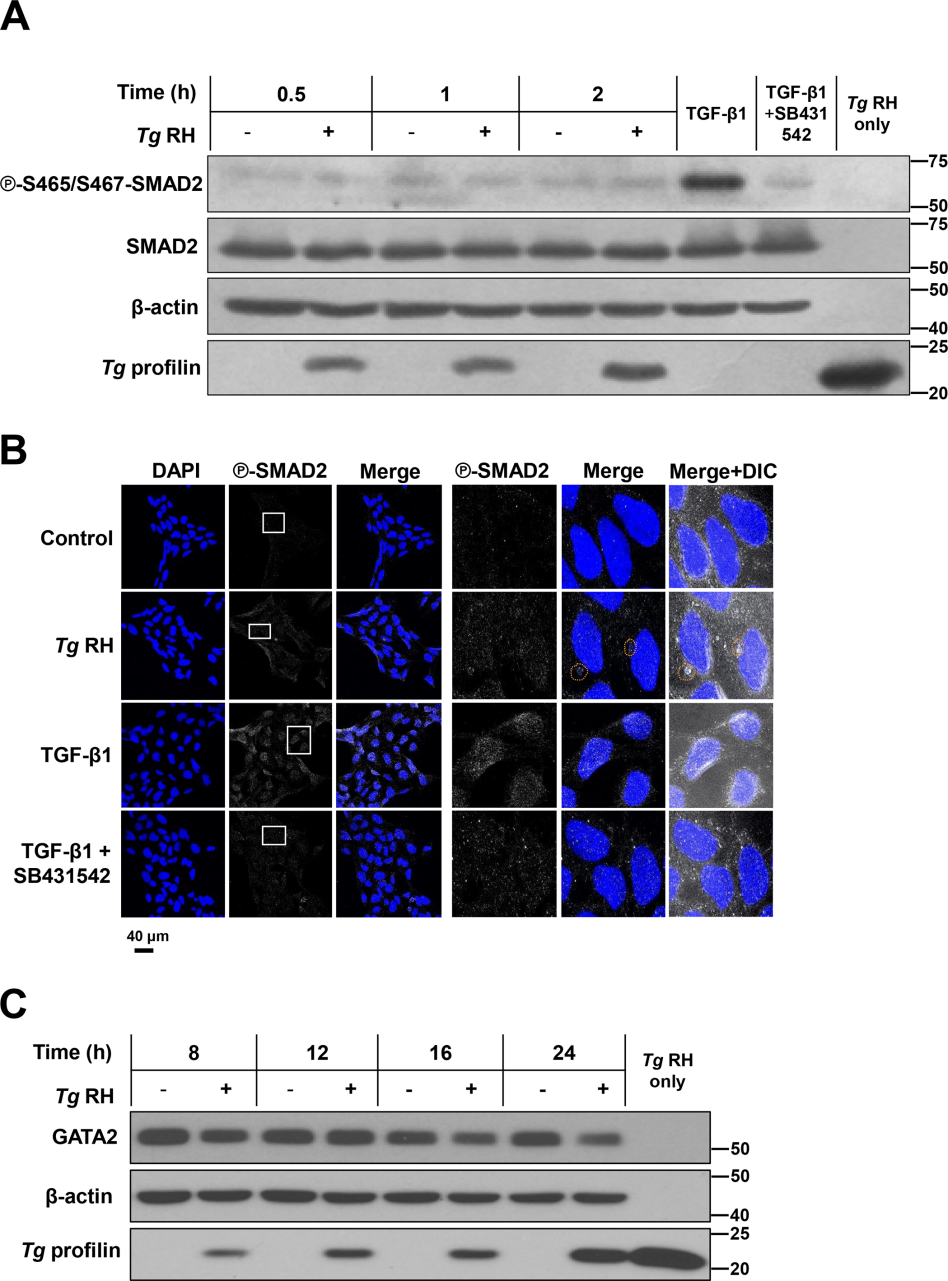
Inhibition of invasion- and migration-related transcripts in *T. gondii*-infected HTR-8/SVneo cells is SMAD2- and GATA2-independent. **(A, B)** Cells were serum-starved and pretreated with 10 μM SB431542 or an equivalent volume of vehicle (i.e., DMSO) for 1 h then stimulated with 5 ng/mL recombinant human TGF-β1 for 2 h, infected with *Tg* RH for 0.5–2 h **(A)** or 2 h **(B)**, or left uninfected (Control) for 2 h. **(A)** Cells were lysed, and samples were processed for western blot analyses to monitor the phosphorylation status (S465/S467) and total levels of SMAD2. β-actin was used as a loading control, and an antibody raised against *T. gondii* profilin-like protein was used to assess infection of HTR-8/SVneo cultures. Total protein extracts from extracellular tachyzoites (“*Tg* only”) were used to control for any cross-reactivity of the antibodies against *T. gondii* proteins. **(B)** Cultures were fixed and processed for confocal immunofluorescence microscopy. Samples were stained with DAPI (shown in blue), used as a nuclear marker, and for phosphorylated SMAD2 at S465/S467 (shown in white). Original magnification (left panels), 4 times-enlarged insets (right panels). Parasitophorous vacuoles (PVs) are outlined with dashed lines to indicate the presence of parasites within infected cells. **(C)** Serum-starved HTR-8/SVneo cultures were infected with *T. gondii* RH strain (*Tg* RH) or left uninfected for the indicated times. Cells were lysed, and samples were processed for western blot analyses to monitor the expression levels of GATA2. **(A–C)** Images are representative of three independent experiments.

The TGF-β1-SMAD2/3 signaling pathway has been shown to repress EVT invasiveness through the inhibition of several transcription factors and invasion-associated genes (Fang et al., 2022; Cheng et al., 2017). Interestingly, phosphorylation and subsequent nuclear translocation of SMAD2 was reported in *T. gondii*-infected macrophages (Damasceno-Sá et al., 2021). Hence, we sought to investigate whether TGF-β1-SMAD2/3 signaling played a role in reduced invasion and expression of associated genes in human EVTs during *T. gondii* infection. To begin testing this hypothesis, we monitored changes in the phosphorylation status of SMAD2 upon infection. As expected, stimulation with recombinant TGF-β1 induced SMAD2 phosphorylation at residues S465/S467 in HTR-8/SVneo cultures (**Figure 3A**), and was blocked upon treatment with the TGF-β1 receptor (ALK4/5/7) inhibitor SB431542 (Inman et al., 2002) without affecting cell viability (**Supplementary Figure S4A**). In stark contrast, RH *T. gondii* tachyzoites failed to upregulate SMAD2 phosphorylation in human EVTs (**Figure 3A**). Consistent with this, nuclear accumulation of SMAD2 was detected in cells stimulated with TGF-β1 and was sensitive to pharmacological blockade of ALK4/5/7 (**Figure 3B**). However, no changes in the subcellular localization of SMAD2 were detected in *T. gondii*-infected HTR-8/SVneo cells.

The transcription factor GATA2 was identified as a negative regulator of the invasion activity and related genes in HTR-8/SVneo cells (Ogoyama et al., 2021). According to our screening, one of the genes negatively targeted by GATA2 was also downregulated in *T. gondii*-infected HTR-8/SVneo cultures (i.e., *MMP14*) (**Figure 1C**). Thus, we set out to elucidate whether *T. gondii*-driven inhibition of *MMP14* in human EVTs was GATA2-dependent. However, western blotting experiments showed that *T. gondii* infection does not increase GATA2 protein levels in HTR-8/SVneo cells compared to uninfected control cultures (**Figure 3C**). Parasite extracts (i.e., isolated from their host cells [“*Tg* only”]) were probed in parallel to rule out potential cross-reactivity of the antibodies tested against parasite proteins (**Figure 3C**). In all, these experiments suggest that *T. gondii*-driven inhibition of invasion in the human trophoblast cell line HTR-8/SVneo occurs through SMAD2- and GATA2-independent mechanisms.

### *Toxoplasma gondii* infection leads to AKT-dependent phosphorylation and nuclear exclusion of FOXO3a and FOXO1 in human trophoblasts

**Figure 4:**
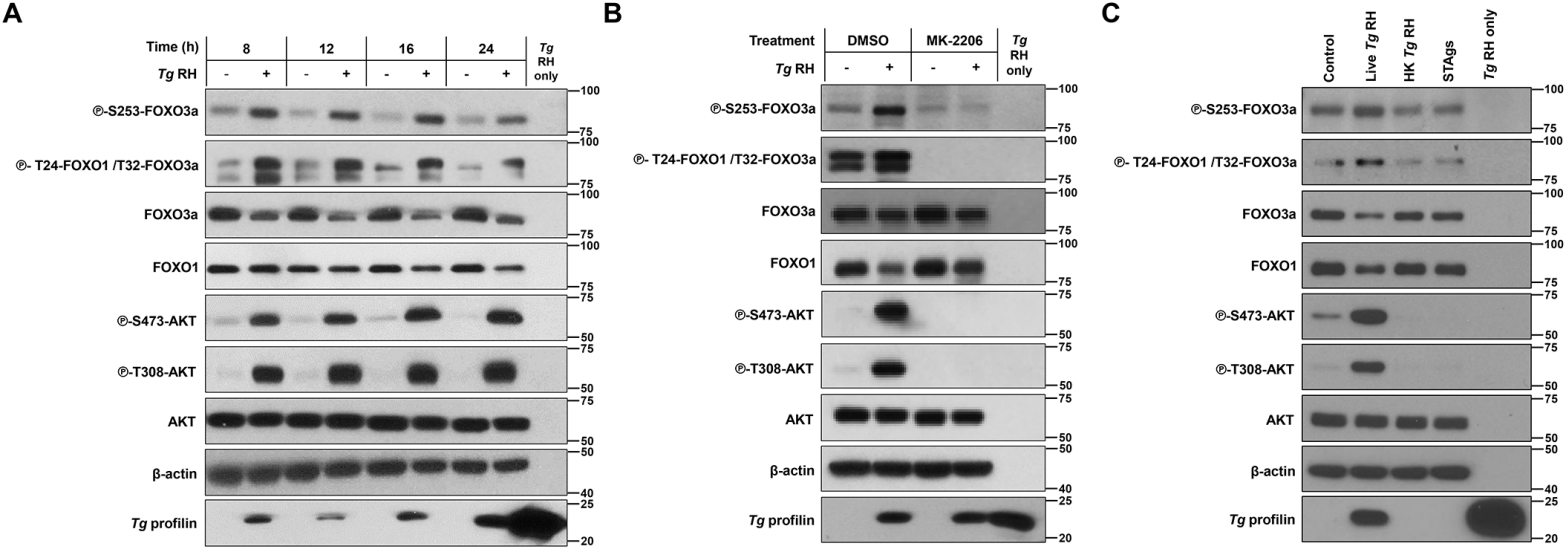
*T. gondii*-driven phosphorylation of FOXO3a and FOXO1 in the human trophoblast cell line HTR-8/SVneo requires live infection and host AKT activity. **(A)** Serum-starved HTR-8/SVneo cultures were infected with *Tg* RH or left uninfected for the indicated times. **(B)** Cells were serum-starved and pretreated with 2 μM MK-2206 or vehicle (i.e., DMSO) for 1 h then infected with *Tg* RH or left uninfected for 24 h. **(C)** After a 1 h serum starvation period, HTR-8/SVneo cultures were inoculated with live or heat-killed (HK) *T. gondii* RH strain (*Tg* RH), treated with 50 μg/mL soluble *T. gondii* antigens (STAgs), or left uninfected and untreated (Control) for 16 h. **(A–C)** Samples were processed for western blot analyses to monitor the phosphorylation and expression levels of indicated proteins. Total amounts of β-actin were used as a loading control, and an antibody raised against *T. gondii* profilin-like protein was used to assess infection of HTR-8/SVneo cultures. Total protein extracts from extracellular tachyzoites (“*Tg* only”) were used to control for any cross-reactivity of the antibodies against *T. gondii* proteins. Data are representative of at least three independent experiments.

In addition to SMAD2 and GATA2, the regulation of gene expression by FOXO3a- and FOXO1 has been associated with EVT invasion and migration (Chen et al., 2022, 2021, 2018). Of note, *T. gondii* has been reported to downregulate FOXO3a and FOXO1 transcriptional activity through their AKT-dependent nuclear exclusion in human fibroblasts and murine macrophages, respectively (Diez et al., 2023; Lee et al., 2022). Thus, we postulated that repression of FOXO3a- and FOXO1-inducible genes and functions also occurred during *T. gondii* infection in human EVTs. To address this, we first monitored changes in the phosphorylation status of AKT, FOXO3a, and FOXO1 by western blotting. Increased and sustained phosphorylation of AKT at residues S473 and T308 was detected in *T. gondii*-infected HTR-8/SVneo cells compared to uninfected control cultures (**Figure 4A**). Accordingly, heightened phosphorylation levels of FOXO3a at AKT-sensitive residues S253 and T32 as well as FOXO1 at residue T24 were observed in infected samples. Of note, the kinetics of FOXO3a and FOXO1 phosphorylation matched closely those observed for AKT. To confirm that *T. gondii*-induced phosphorylation of FOXO3a and FOXO1 was mediated by AKT, HTR-8/SVneo cells were treated with MK-2206, a pan-AKT inhibitor (Hirai et al., 2010), or with DMSO (vehicle). AKT phosphorylation at S473 and T308 was abrogated in the presence of MK-2206 regardless of infection status (**Figure 4B**) without affecting cell viability (**Supplementary Figure S4B**). Importantly, pharmacological inhibition of AKT prevented FOXO3a phosphorylation at S253 and T32 and FOXO1 phosphorylation at T24 in *T. gondii*-infected HTR-8/SVneo cultures (**Figure 4B**) (Diez et al., 2023). Furthermore, modulation of FOXO3a and FOXO1 phosphorylation required live parasite infection as no changes were observed following treatment with HK parasites or STAgs (**Figure 4C**).

**Figure 5:**
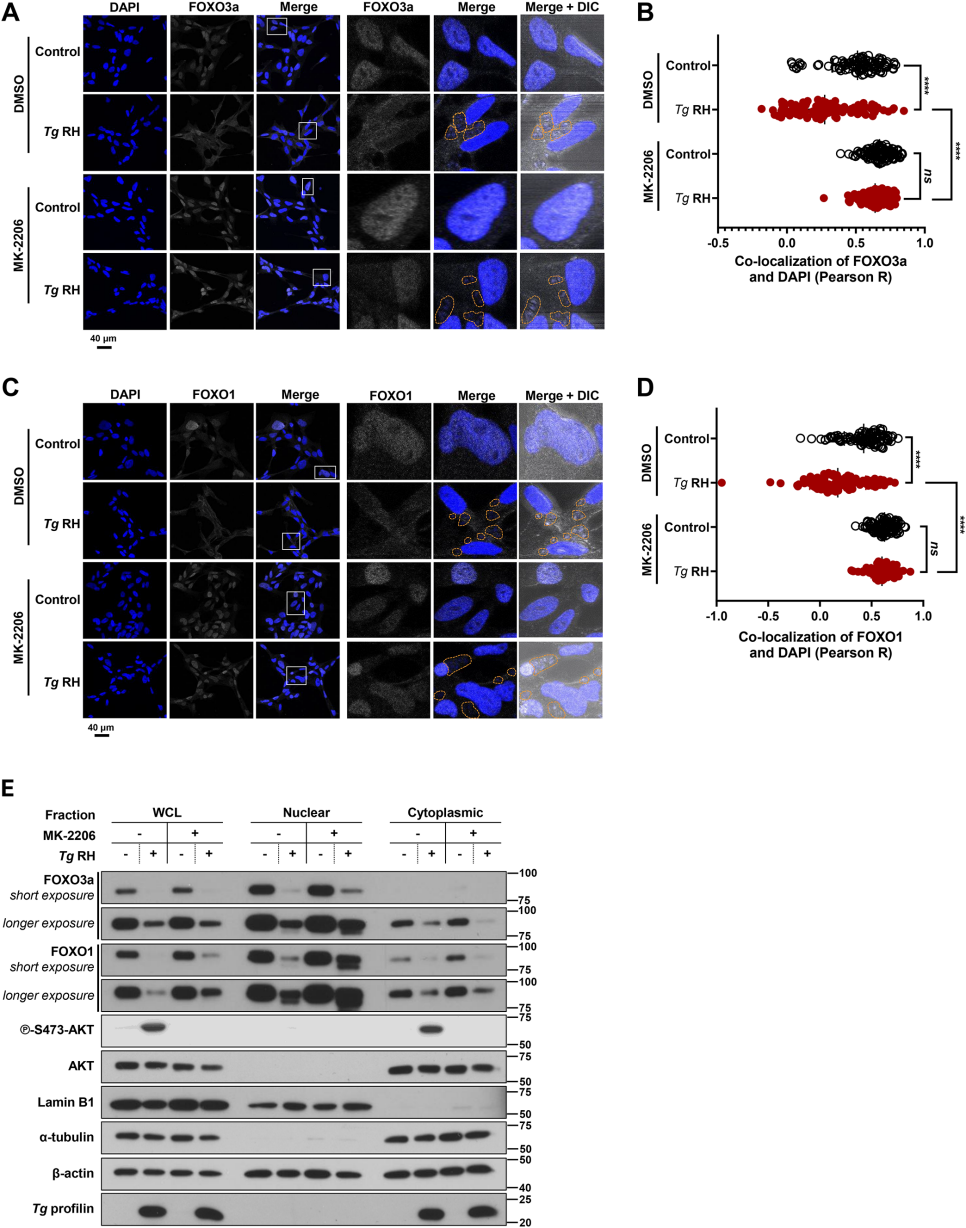
*T. gondii* infection promotes AKT-sensitive nuclear export of FOXO3a and FOXO1 in the human trophoblast cell line HTR-8/SVneo. Serum-starved HTR-8/SVneo cultures were pretreated with 2 μM MK-2206 or an equivalent volume of vehicle (i.e., DMSO) for 1 h then infected with *Tg* RH or left uninfected for 16 h. **(A, C)** Cultures were fixed and processed for confocal immunofluorescence microscopy. Samples were stained with DAPI (shown in blue), used as a nuclear marker, and for FOXO3a **(A)** or FOXO1 **(C)** (shown in white). Original magnification (left panels), 4 times-enlarged insets (right panels). PVs are outlined with dashed lines to indicate the presence of parasites within infected cells. Images are representative of two independent experiments. Co-localization of FOXO3a **(B)** or FOXO1 **(D)** and DAPI was quantified using the Pearson R coefficient. **(E)** Samples were processed for whole cell lysates (WCL), nuclear, and cytoplasmic fraction for western blot analyses to monitor the phosphorylation and expression levels of indicated proteins. Total amounts of β-actin were used as a loading control, Lamin B1 was used as a control of nuclear fraction, α-tubulin was used as a cytoplasmic control, and an antibody raised against *T. gondii* profilin-like protein was used to assess infection of HTR-8/SVneo cultures. **(A–D)** Data are representative of two independent experiments (n = 2) in which 50 cells were analyzed in different fields of view. Each data point represents Pearson R coefficient of one cell. Statistical significance was determined using a two-way ANOVA, where ****P < 0.0001; *ns* not significant. **(E)** Data are representative of three independent experiments.

Based on our data showing *T. gondii*-inducible AKT-sensitive phosphorylation of FOXO3a and FOXO1 in EVTs (**Figures 4A–C**), we assessed the subcellular localization of these transcription factors in HTR-8/SVneo cells following *T. gondii* infection. Under serum-deprived conditions, FOXO3a and FOXO1 were detected mostly in the nucleus in uninfected control cells (**Figures 5A–D**), consistent with low phosphorylation of FOXO3a and FOXO1 at AKT-sensitive residues (**Figures 4A–C**). Conversely, FOXO3a and FOXO1 were exported from the host cell nucleus upon *T. gondii*-infection (**Figures 5A, C**, respectively) in an MOI-dependent fashion (**Supplementary Figures S5A, B**, respectively). Significant differences in FOXO3a and FOXO1 subcellular localization were highlighted by their decreased co-localization coefficients with nuclear staining (**Figures 5B, D**, respectively). As expected, treatment of HTR-8/SVneo cultures with the pan-AKT inhibitor MK-2206 prevented *T. gondii*-driven changes in FOXO3a and FOXO1 subcellular localization (**Figures 5A–D**, respectively). In line with immunofluorescence and confocal microscopy data, western blot analyses of nuclear and cytoplasmic protein extracts confirmed AKT-sensitive nuclear exclusion of FOXO3a and FOXO1 in *T. gondii*-infected HTR-8/SVneo cells (**Figure 5E**). Taken together, these results indicate that *T. gondii* induces AKT-dependent phosphorylation and nuclear export of FOXO3a and FOXO1 in human EVTs.

### FOXO3a and FOXO1 inactivation is involved in the inhibition of invasion and migration in *T. gondii*-infected trophoblasts

**Figure 6:**
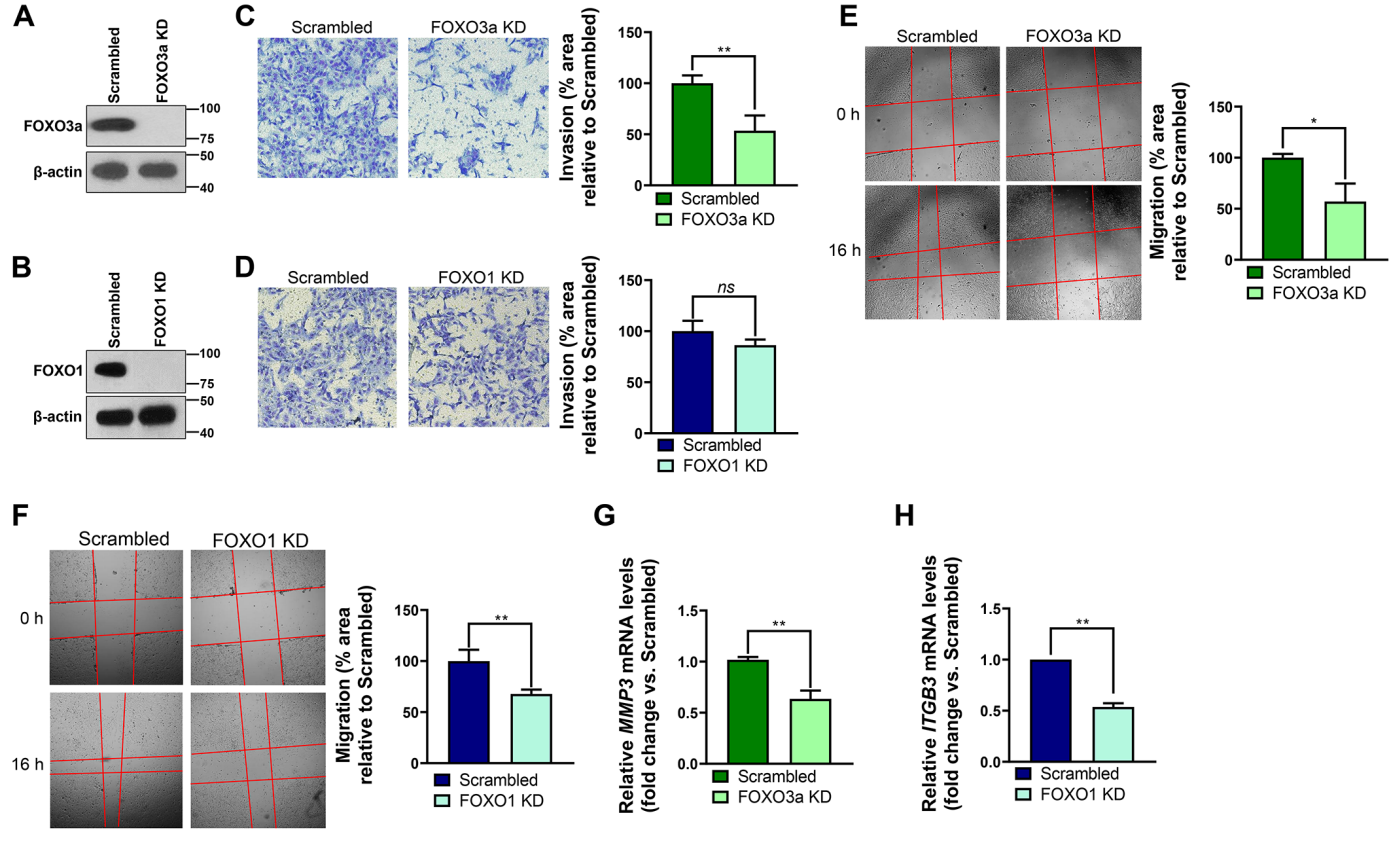
FOXO3a and FOXO1 are required for invasion, migration, and transcription of related genes in the human trophoblast cell line HTR-8/SVneo. FOXO3a **(A)** and FOXO1 **(B)** protein levels were monitored by western blotting in HTR-8/SVneo cell lines transduced with scrambled, FOXO3a or FOXO1 shRNAs (i.e., Scrambled, FOXO3a knocked-down [KD], and FOXO1 KD, respectively). Total amounts of β-actin were used as a loading control. Images are representative of three independent experiments. **(C, D)** Matrigel-based invasion assays were carried out for 16 h in serum-starved Scrambled, FOXO3a KD **(C)**, and FOXO1 KD **(D)** HTR-8/SVneo cell lines. Shown here are representative images of three independent experiments (left panels). Quantification of cell invasion (right panels). **(E, F)** Wound-healing assays were performed to monitor migration activity of serum-starved Scrambled, FOXO3a KD **(E)**, and FOXO1 KD **(F)** HTR-8/SVneo cell lines. Representative images of three independent experiments (left panels). Quantification of cell migration (right panels). **(G, H)** Relative amounts of indicated invasion- and migration-related transcripts were quantified in serum-starved Scrambled, FOXO3a KD **(G)**, and FOXO1 KD **(H)** HTR-8/SVneo cell lines by RT-qPCR. Relative expression was normalized to *HPRT1*. **(C-H)** Values are presented as mean [SD] calculated from three independent experiments (n = 3). Statistical significance was determined using a two-tailed independent Student’s *t-*test, where **P < 0.01; *P < 0.05; *ns* not significant.

Next, we set out to elucidate whether parasite-driven repression of FOXO3a and FOXO1 contributed to reduced invasion and migration activities in *T. gondii*-infected human trophoblasts. To address this, we first carried out invasion and migration assays in HTR-8/SVneo cell lines knocked down (KD) in FOXO3a and FOXO1 (**Figures 6A, B**, respectively) generated by lentiviral transduction of shRNAs. Consistent with previous reports (Chen et al., 2021, 2022), invasion and migration properties were significantly downregulated in FOXO3a KD EVTs compared with Scrambled control cultures (**Figures 6C, E**, respectively). Unlike FOXO3a, FOXO1 appeared to be dispensable for HTR-8/SVneo invasiveness, as no differences were detected between Scrambled and FOXO1 KD cell lines (**Figure 6D**). However, migration activity was significantly diminished in HTR-8/SVneo cells expressing reduced levels of FOXO1 (**Figure 6F**), in line with a previous report (Chen et al., 2018). Importantly, differences in invasion and migration detected between Scrambled and FOXO3a or FOXO1 KD HTR-8/SVneo cell lines were not due to defects in cell viability and proliferation rates (**Supplementary Figures S6A-D**).

**Figure 7:**
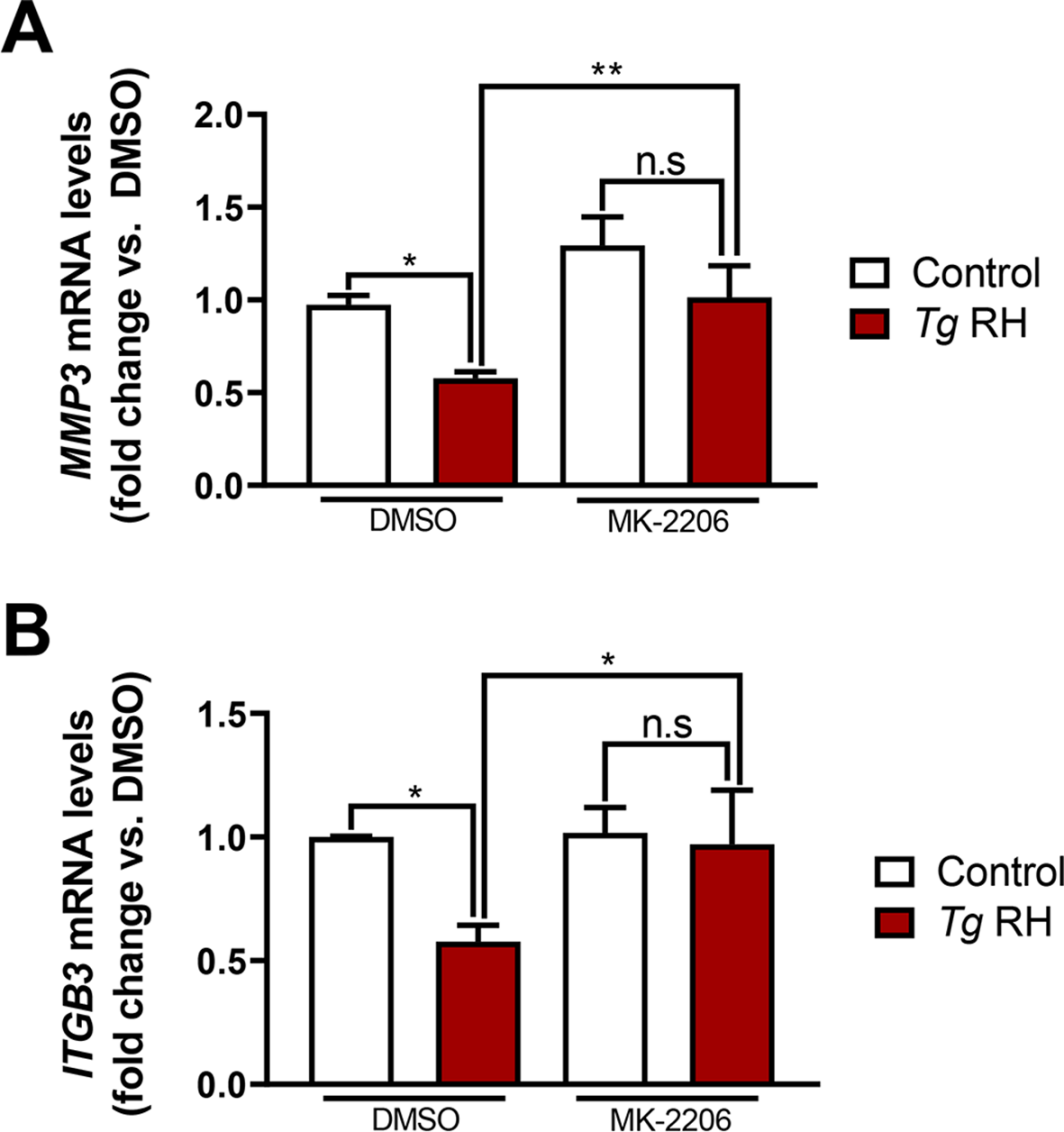
Downregulation of *MMP3* and *ITGB3* mRNA expression in *T. gondii*-infected HTR-8/SVneo cells is AKT dependent. Serum-starved HTR-8/SVneo cultures were pretreated with 2 μM MK-2206 or vehicle (i.e., DMSO) for 1 h then infected with *Tg* RH or left uninfected for 16 h Relative amounts of *MMP3***(A)** and *ITGB3***(B)** were quantified by RT-qPCR. Relative expression was normalized to *HPRT1*. Values are presented as mean [SD] calculated from three independent experiments (n = 3). Statistical significance was determined using a two-way ANOVA, where **P < 0.01; *P < 0.05; *ns* not significant.

**Figure 8:**
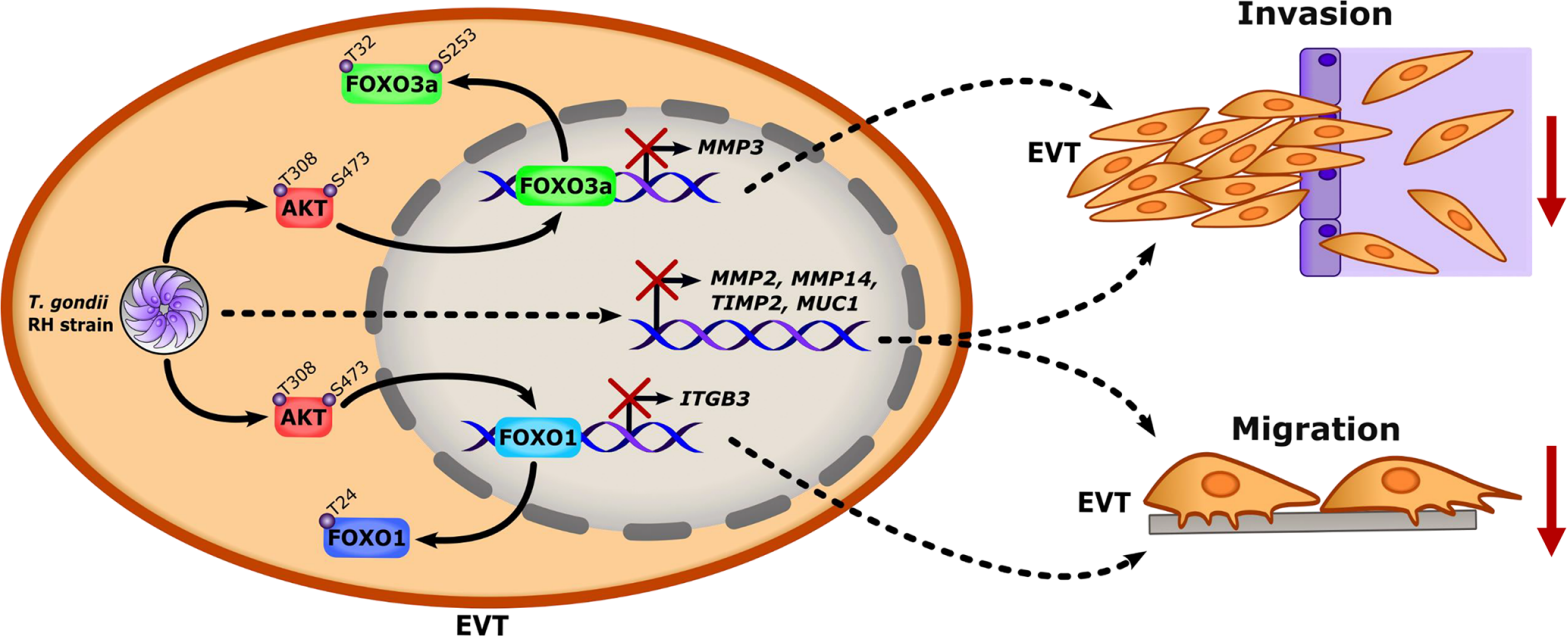
*T. gondii* infection represses invasion and migration activities in HTR-8/SVneo cells through FOXO3a/1-dependent and -independent gene expression (Proposed model). Upon infection of human EVTs, *T. gondii* activates the host kinase AKT which in turn phosphorylates the transcription factors FOXO3a at S253 and T32, and FOXO1 at T24. These events lead to AKT-sensitive nuclear exclusion of FOXO3a and FOXO1, and reduced expression of FOXO3a- and FOXO1-dependent genes (i.e. *MMP3* and *ITGB3*, respectively). In addition, to *MMP3* and *ITGB3*, *T. gondii* down-regulates transcription of other invasion- and migration-related genes (i.e. *MMP2*, *MMP14*, *TIMP2*, and *MUC1*) through yet-to-be-defined mechanisms. Collectively, these data indicate that invasion and migration activities are reduced in *T. gondii*-infected EVTs in a FOXO3a/1-dependent and -independent fashion.

After having confirmed that FOXO3a and FOXO1 are required for HTR-8/SVneo cell invasiveness and/or migration, we then monitored whether *T. gondii*-driven inhibition of EVT invasion- and migration-related genes (**Figure 1C**) was dependent on FOXO3a and/or FOXO1 repression. RT-qPCR experiments showed reduced *MMP3* mRNA expression in FOXO3a KD HTR-8/SVneo cells compared with Scrambled control cultures (**Figure 6G**). Similarly, accumulation of *ITGB3* transcripts was diminished in an HTR-8/SVneo cell line expressing reduced levels of FOXO1 (**Figure 6H**). Based on our data from reverse-genetics experiments (**Figures 6A–H**) along with results showing that pharmacological blockade of AKT prevented FOXO3a and FOXO1 nuclear exclusion despite *T. gondii* infection (**Figures 5A–D**, respectively), we formulated the hypothesis that parasite-driven AKT-sensitive inhibition of FOXO3a and FOXO1 activity contributes to repression of invasion- and migration-associated genes *MMP3* and *ITGB3* in *T. gondii*-infected EVTs. In line with this notion, RH *T. gondii* tachyzoites failed to downregulate *MMP3* and *ITGB3* mRNA levels in HTR-8/SVneo cells treated with the pan-AKT inhibitor MK-2206 (**Figures 7A, B**, respectively). Taken together, these data suggest that *T. gondii* inhibits invasion and migration properties in the human trophoblast cell line HTR-8/SVneo through FOXO3a/FOXO1-dependent and -independent mechanisms (**Figure 8**).

## Discussion

Primary *T. gondii* infection during the first trimester of gestation is associated with high risk of spontaneous abortion, stillbirth, premature birth, and fetal growth restriction (Borges et al., 2019). It is well-documented that *T. gondii*-driven dysregulation of maternal immune responses in the placental microenvironment contributes to such poor pregnancy outcomes (Borges et al., 2019; Liu et al., 2018; Zhan et al., 2018). However, the cellular and molecular mechanisms involved are not fully understood. An *ex vivo* study using human placental explants from first trimester pregnancies revealed that EVTs are quite permissive to *T. gondii* infection in contrast to syncytiotrophoblast (Robbins et al., 2012). Similar results were observed *in vitro* following infection of human EVT and villous trophoblast cell lines with *T. gondii* (Almeida et al., 2019). Interestingly, Wang and colleagues recently reported a decrease in EVT invasion and migration activities when co-cultured with human macrophages pre-treated with *T. gondii* antigens (Wang et al., 2024), suggesting indirect parasite-driven EVT dysregulation. Aberrant EVT migration and invasion of the decidua have deleterious consequences during the first trimester of pregnancy (Kudo and Sugimoto, 2024); however, no direct link had been established between *T. gondii* infection and alteration of EVT functions required for normal placentation and embryo implantation. Herein, using cell imaging in combination with pharmacological and reverse genetics approaches in the human trophoblast cell line HTR-8/SVneo, we demonstrate that *T. gondii* infection directly downregulates invasiveness and migration of HTR-8/SVneo cells by repressing FOXO1- and FOXO3a-dependent and -independent transcriptional programs. Our data show that *T. gondii* infection decreases the expression of genes encoding several MMPs (i.e., *MMP2*, *MMP14*, and *MMP3*) in HTR-8/SVneo cells. These enzymes are essential for extracellular matrix degradation, a key step in EVT invasiveness and migration activities (Zhu et al., 2012). We also confirmed *T. gondii*-driven inhibition of transcripts responsible for the synthesis of integrin β3 and Mucin 1 (*ITGB3* and *MUC1*, respectively) two proteins required for EVT adhesion and migration to the maternal decidua (Chen et al., 2018; Thirkill et al., 2007). Interestingly, in addition to type I RH *T. gondii* strain, we observed that a type II strain (i.e., ME49) is able to reduce migration of HTR-8/SVneo cells without affecting their invasiveness (**Supplementary Figure S7**). These data warrant further investigation to determine whether *T. gondii*-driven modulation of human EVT functions is strain-dependent and identify potential strain-specific regulatory mechanisms.

*In vivo* studies using mice harboring a specific deletion for *Foxo3* or *Foxo1* in the uterine compartment have demonstrated that these the transcription factors are essential for successful endometrial decidualization and embryo implantation during early pregnancy (Long et al., 2019; Vasquez et al., 2018). Moreover, genetic ablation of *FOXO3* or *FOXO1* showed their requirement for invasion and migration activities and associated transcriptional programs in human EVTs (Chen et al., 2022, 2021, 2018). Of note, alterations in FOXO3a and FOXO1 expression and activity have been associated with preeclampsia (Chen et al., 2021, 2020; Zhang et al., 2022a; Chen et al., 2018; Akkaya Firat et al., 2025), a pregnancy-related complication characterized by impaired EVT invasion and migration (Chen et al., 2021, 2020, 2018). For instance, lower FOXO3a expression and increased phosphorylation at the AKT-sensitive residue S253 were detected in placental tissues of preeclampsia patients when compared to samples isolated from healthy pregnant women (Chen et al., 2021). Similarly, FOXO1 was reported to be poorly expressed in the placenta of preeclampsia patients in comparison to the control group (Chen et al., 2020; Sheridan et al., 2015; Zhang et al., 2022a; Chen et al., 2018). Furthermore, a significant decrease in FOXO1 serum concentrations was correlated with the onset of preeclampsia (Akkaya Firat et al., 2025). In line with these findings, we observed that *T. gondii* infection induces AKT-sensitive phosphorylation and nuclear exclusion of FOXO3a and FOXO1 in HTR-8/SVneo cells. In addition, we detected a marked reduction in FOXO3a and FOXO1 protein expression upon infection. These data agree with previous work showing that AKT-dependent FOXO3a and FOXO1 phosphorylation and translocation to the cytosolic compartment induces binding of the chaperone protein 14-3–3 and proteasomal degradation (Biggs et al., 1999; Calissi et al., 2021). Future investigation using co-immunoprecipitation assays and proteasome inhibitors will provide insight into the mechanistic basis for decreased FOXO3a and FOXO1 levels in *T. gondii*-infected EVTs.

Our reverse genetics experiments combined with pharmacological blockade of AKT indicate that *T. gondii* downregulates the expression of migration- and -invasion associated genes *MMP3* and *ITGB3* via AKT-dependent phosphorylation and nuclear exclusion of FOXO3a and FOXO1, respectively. Even though a potential link between congenital toxoplasmosis and preeclampsia has been explored, a clear correlation remains to be established (Liu et al., 2018; Alvarado-Esquivel et al., 2014). Thus, based on our data along with previous reports, it is tempting to speculate that parasite-driven repression of FOXO3a- and FOXO1-regulated transcriptional programs and biological processes in EVTs contribute to pregnancy-related pathologies, including preeclampsia. Further investigation using *ex vivo* human placental models combined with experimental models of congenital toxoplasmosis in mice deficient for *Foxo3* and *Foxo1* in the uterine compartment will help shed light on this matter.

Among TORCH pathogens, only *T. gondii* and human cytomegalovirus (HCMV) have been shown to disrupt host cell functions by modulating the activity of FOXO family members (Hale et al., 2020; Chen et al., 2022; Diez et al., 2023; Lee et al., 2022). For instance, HCMV infection induces FOXO3a nuclear translocation which is crucial for immediate early viral gene expression and HCMV reactivation in fibroblasts (Hale et al., 2020; Chen et al., 2022). In stark contrast, *T. gondii* inhibits FOXO3a-regulated transcriptional programs related to autophagy via AKT-dependent nuclear export of FOXO3a in human fibroblasts (Diez et al., 2023). Similarly, AKT-inducible phosphorylation and cytoplasmic accumulation of FOXO1 were reported in *T. gondii*-infected macrophages (Lee et al., 2022). Despite this evidence, dysregulation of FOXO transcriptional activity by TORCH pathogens, including *T. gondii*, had not been investigated in trophoblasts. Confirming and extending previous studies in fibroblasts and macrophages (Diez et al., 2023; Lee et al., 2022), we observed that *T. gondii* inhibits transcription of invasion and migration-related genes associated FOXO3a and FOXO1 activity in an AKT-dependent fashion in the human trophoblast cell line HTR-8/SVneo. Similar to *T. gondii*, other TORCH pathogens, such as *Listeria monocytogenes*, preferentially infect EVTs (Robbins et al., 2010). Interestingly, upregulation of AKT phosphorylation was reported in HCMV-infected human EVTs (Lin et al., 2020). Hence, future investigation is required to determine whether in addition to *T. gondii*, inactivation of FOXO3a and/or FOXO1 via AKT-sensitive phosphorylation by other TORCH pathogens, contributes to dysregulation of EVT functions and poor pregnancy outcomes.

Reverse genetics approaches combined with transcriptional profiling studies carried out in the human cell line HTR-8/SVneo identified FOXO3a as a central regulator of EVT migration and invasion activities (Chen et al., 2022, 2021). More specifically, RNA-seq experiments in WT and FOXO3a KD cells identified *ICAM1*, *PTGS2*, and *MMP9* among the top migration- and invasion-associated genes under transcriptional control of FOXO3a in human EVTs (Chen et al., 2022, 2021). Surprisingly, we did not detect a significant reduction in *ICAM1*, *PTGS2*, and *MMP9* mRNA levels in FOXO3a KD versus control WT HTR-8/SVneo cells. This discrepancy might be related to differences in the experimental design utilized in both studies, including target identification and validation methods (i.e., RNA-seq versus RT-qPCR), cell culture conditions, time of incubation, lysis buffer for sample collection, etc. Even though *MMP3* had not been previously reported as a transcriptional target of FOXO3a, bioinformatics approaches identified three consensus binding sites for FOXO3a in the *MMP3* promoter, suggesting a functional interaction (Samatar et al., 2002). In line with this *in silico* analysis, our RT-qPCR experiments revealed a significant decrease in *MMP3* mRNA abundance in FOXO3a KD HTR-8/SVneo cultures when compared to WT control cells. Of note, inhibition of *MMP3* gene expression was phenocopied in WT HTR-8/SVneo cells infected with *T. gondii* and was reversed by pharmacological blockade of AKT. These findings along with our microscopy data showing that *T. gondii* drives AKT-dependent FOXO3a phosphorylation and nuclear exclusion, suggest that *MMP3* downregulation in *T. gondii*-infected EVTs is due to the inhibition of FOXO3a transcriptional activity via AKT.

It is well-documented that MMP3 levels increase in various types of cancer which facilitates tumor cell invasion and metastasis (Phromnoi et al., 2009; Liang et al., 2021; Seccareccia et al., 2014). Although less investigated, a growing body of work suggests that MMP3 is also involved in EVT invasion and normal pregnancy outcomes (Husslein et al., 2009; Demir-Weusten et al., 2007; Reister et al., 2006; Williams et al., 2011). Immunofluorescence analyses of first trimester placental tissues combined with western blot data of isolated primary placental cells revealed that MMP3 is mostly expressed and secreted by villous trophoblasts and EVTs (Husslein et al., 2009). In addition, MMP3 was detected in EVTs of the basal plate in human term placental samples, specifically in EVTs that are in direct contact with maternal cells, suggesting a role for MMP3 during labor (Demir-Weusten et al., 2007). Importantly, a reduction in MMP3 expression in EVTs surrounding spiral arteries was reported in patients with severe preeclampsia as compared to healthy controls (Reister et al., 2006). Furthermore, treatment with folic acid was shown to enhance human EVT invasion, an effect that was associated with increased secretion of MMP3 and MMP2 in human placental explants (Williams et al., 2011). Thus, based on previous reports and our findings, it is conceivable that *T. gondii*-driven repression of MMP3 is partially responsible for reduced EVT invasion activity and pregnancy complications associated with congenital toxoplasmosis. Future work will shed light on this matter.

The cell surface glycoprotein integrin β3 binds to diverse extracellular matrix molecules (e.g., fibrinogen, collagen, fibronectin, etc.) and triggers intracellular signals involved in cell invasion and migration (Lei et al., 2011). Reduced integrin β3 (Chen et al., 2018) and FOXO1 (Chen et al., 2020; Sheridan et al., 2015; Zhang et al., 2022a; Chen et al., 2018) levels were detected in placental samples isolated from preeclampsia patients when compared to healthy controls. Furthermore, mechanistic studies revealed that FOXO1-dependent transcription of the integrin β3-encoding gene *ITGB3* is required for HTR-8/SVneo cell migration and adhesion activities (Chen et al., 2018). The authors also reported that FOXO1 transcriptional activity, integrin β3 expression, and migration properties in EVTs are negatively regulated by the kinase AKT (Chen et al., 2018). In line with these findings, we observed that either *T. gondii* infection or genetic ablation of FOXO1 reduces *ITGB3* mRNA accumulation and migratory activity in HTR-8/SVneo cells. Moreover, we demonstrated that *T. gondii-*driven activation of AKT triggers FOXO1 nuclear exclusion and downregulates *ITGB3* mRNA levels in infected HTR-8/SVneo cultures. This set of experiments along with previous reports support the notion that inhibition of FOXO1 transcriptional activity via AKT leads to a decrease in *ITGB3* gene expression and contributes to reduced migration properties in *T. gondii*-infected EVTs. Further investigation is required to confirm these findings *in vivo*.

Our reverse genetics experiments provide evidence that reduced expression of FOXO3a or FOXO1 downregulates but does not completely abrogate the invasive and migratory properties of human EVTs. Consistent with this, among the invasion- and migration-associated transcripts whose expression diminished in *T. gondii*-infected EVTs (i.e., *MMP2*, *MMP3*, *MMP14*, *TIMP2*, *MUC1*, and *ITGB3*), we only identified *MMP3* and *ITGB3* as transcriptional targets of FOXO3a and FOXO1, respectively. These data indicate that *T. gondii* also alters migration, invasion, and related transcriptional programs thorough FOXO3a- and FOXO1-independent mechanisms. For instance, the TGF-β1-SMAD2/3 signaling pathway, which is activated by *T. gondii* infection in macrophages (Damasceno-Sá et al., 2021), was reported to reduce invasiveness by preventing invasion-related gene expression (i.e., connective tissue growth factor and kisspeptin) in of HTR-8/SVneo cells (Fang et al., 2022; Cheng et al., 2017). Similarly, the transcription factor GATA2 was described as a negative regulator of trophoblast invasion through *MMP14* repression (Ogoyama et al., 2021). Surprisingly, our data revealed that the phosphorylated form of SMAD2 is not translocated to the nucleus and GATA2 expression does not increase in *T. gondii*-infected HTR-8/SVneo cells. Therefore, it is unlikely that *T. gondii*-driven repression of HTR-8/SVneo invasion is controlled by the TGF-β1-SMAD2/3 or GATA2 signaling pathways.

MMP2 is one of the most critical MMPs involved in EVTs invasion (Staun-Ram et al., 2004; Seval et al., 2004; Li et al., 2015). The *MMP2* gene was identified as a FOXO3a transcriptional target in smooth muscle cells in response to NOX4 and urotensin-II signaling (Diebold et al., 2011). However, in our HTR-8/SVneo FOXO3a KD model, *MMP2* mRNA accumulation did not appear to be regulated via FOXO3a. Nevertheless, we cannot exclude the possibility that *T. gondii* infection inhibits *MMP2* expression through alternative mechanisms. Changes in mRNA levels observed in our screening could also stem from post-transcriptional regulatory mechanisms such as miRNA activity, which has been reported during *T. gondii* infection in human placental explants (Medina et al., 2020). Of note, miR-346 and miR-582-3p have been shown to reduce *MMP2* mRNA expression and MMP2 activity as well as invasion and migration in the HTR-8/SVneo cell line (Su et al., 2017). Hence, miRNA-dependent repression of migration and invasion activities in *T. gondii*-infected EVTs, including but not limited to the downregulation of *MMP2*, deserves further investigation.

In addition to *MMP2*, we identified other FOXO3a-/FOXO1-independent genes whose downregulation during *T. gondii* infection may also contribute to impaired invasion and migration activities in EVTs (i.e., *MMP14*, *TIMP2*, and *MUC1*). In support of this notion, MMP14 and TIMP2 are known to form a complex that facilitates the activation of pro-MMP2 (Nishida et al., 2008). Accordingly, a decrease in both MMP14 and TIMP2 expression has been associated with impaired EVT invasion (Wang et al., 2014; Ogoyama et al., 2021; Onogi et al., 2011). Furthermore, genetic ablation of MMP3 was shown to inhibit the enzymatic activity of MMP2 in human umbilical vein endothelial cells (Lee et al., 2008). These previous studies along with our findings warrant further investigation to determine whether in addition to transcriptional repression, *T*. *gondii* indirectly inhibits MMP2 by blocking *MMP3* gene expression, as well as the activity of other migration- and invasion-related proteins in EVTs, through post-translational regulatory mechanisms.

In sum, we report a novel mechanism whereby *T. gondii* prevents migration and invasion activities in infected human trophoblast cell line HTR-8/SVneo that involves the inhibition of FOXO3a/1-dependent and -independent transcriptional mechanisms (**Figure 8**). Given that we only carried out a screening of a subset of invasion- and migration associated genes, future work using reverse and forward genetics combined with multi-omics approaches (i.e., RNA-seq, miR-seq, ChIP-seq, and proteomics) is likely to provide further insights into EVT dysregulation during *T. gondii* infection via FOXO3a/1-dependent- and independent transcriptional repression and alternative mechanisms, such as miRNAs and post-translational modifications. Importantly, this study highlights the disruption of central EVT functions during *T. gondii* infection *in vitro* and warrant further investigation using *ex vivo* and *in vivo* models, and primary cells to determine whether defects in EVT migration and invasion contribute to the pathogenesis of congenital toxoplasmosis.

## Data Availability

The original contributions presented in the study are included in the article/**Supplementary Material**. Further inquiries can be directed to the corresponding author.
