# Supplementary material for: Toxoplasma gondii infection inhibits invasion and migration of human extravillous trophoblasts through dysregulation of FOXO1- and FOXO3a-dependent and -independent mechanisms
Source: Front Cell Infect Microbiol. 2025 Dec 10;15:1651142. doi: 10.3389/fcimb.2025.1651142 (PMC12727940; doi:10.3389/fcimb.2025.1651142)
Supplement: Supplementary file 1 [file DataSheet1.pdf]

Supporting Information

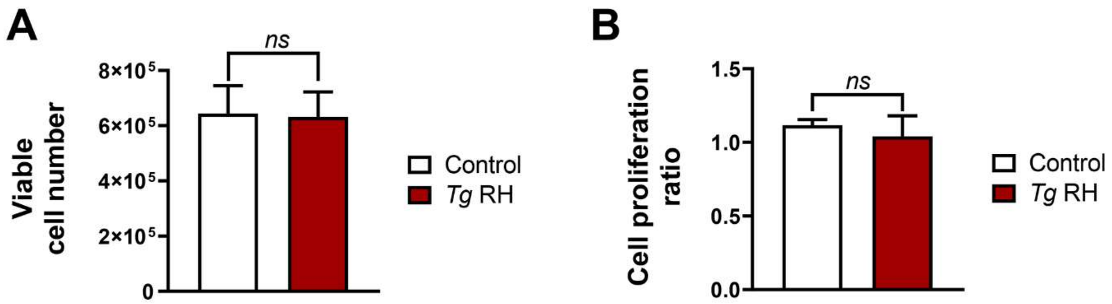

Supplementary Figure 1. *T. gondii* infection does not affect viability and proliferation rates of human trophoblast cell line HTR-8/SVneo. Cells were incubated in serum-free media 1 h before infection with *Tg* RH strain (MOI 5:1) or left uninfected (Control). **(A)** Number of live cells was quantified at 16 h.p.i. (**B)** Cell proliferation ratio was calculated by comparing live cell counts at 0 h and 16 h post-scratch wound. (**A-B**) Values are presented as mean [SD] calculated from three independent experiments (n = 3). Statistical significance was determined using a two-tailed independent Student's *t*-test. *ns*, not significant.

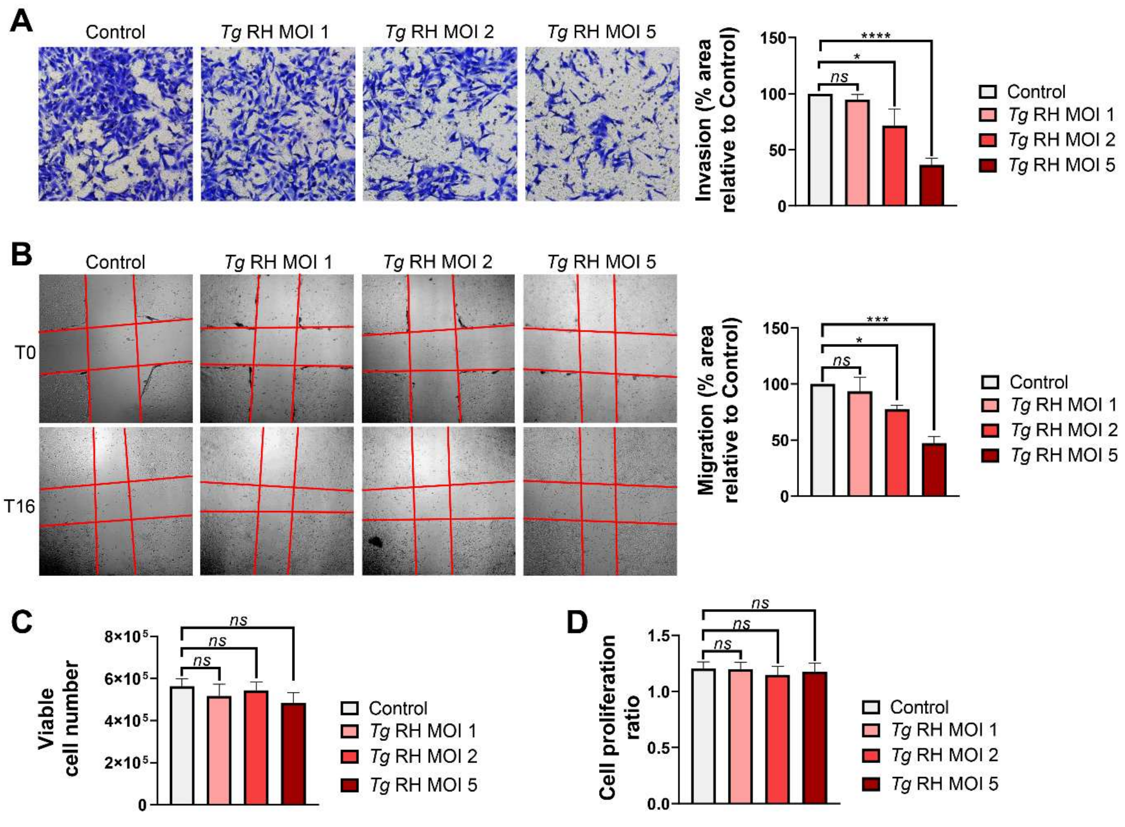

Supplementary Figure 2. ***T. gondii*** **downregulates HTR-8/SVneo cell invasion and migration in an MOI- dependent manner. (A-D)** Cells were incubated in serum-free media 1 h before infection with the indicated MOI of *T. gondii* RH strain or left uninfected (Control). **(A)** Collagen-based matrix invasion was monitored at 16 hours post-infection (h.p.i). Shown here are representative images of three independent experiments (left panel) and quantification of cell invasion (right panels). **(B)** Cell migration was assessed by wound-healing assays. Shown here are representative images of three independent experiments (left panels) and quantification of cell migration (right panel). **(C)** Number of live cells was quantified at 16 h.p.i. (**D)** Cell proliferation ratio was calculated by comparing live cell counts at 0 h and 16 h post-scratch wound. **(A-D)** Values are presented as mean [SD] calculated from three independent experiments (n = 3). The statistical significance was determined a one-way

ANOVA followed by post-hoc Tukey's test, where **** $P < 0.0001$; ***$P < 0.001$; * $P < 0.5$; *ns* not significant.

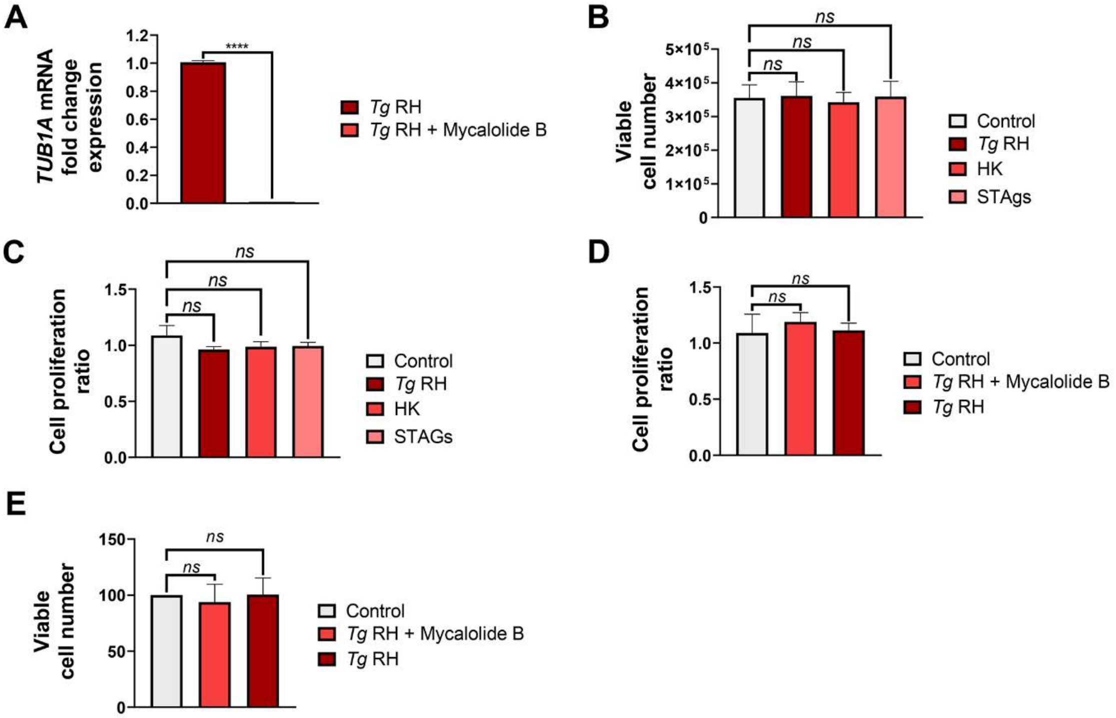

Supplementary Figure 3. HTR-8/SVneo cell viability and proliferation rates are not affected by treatment with soluble *T. gondii* antigens or incubation with heat-killed or mycalolide-pretreated parasite cultures. Serum-starved HTR-8/SVneo cultures were incubated with DMSO- or mycalolide B-pretreated *Tg* RH tachyzoites (**A**, **D-E**), inoculated with live or heat-killed (HK) *Tg* RH, treated with 50 µg/mL soluble *T. gondii* antigens (STAgs) **(B-C)**, or left uninfected and untreated (Control) (**A-E**) for 16 h. **(A)** Relative amounts of *T. gondii TUB1A* mRNA were quantified by RT-qPCR. Relative expression was normalized to *HPRT1*. **(B, E)** Number of live cells was quantified at 16 h.p.i. / post-treatment. (**C, D)** Cell proliferation ratio was calculated by comparing cell counts at 0 h and 16 h post-scratch-wound. (A-E) Values are presented as mean [SD] calculated from three independent experiments (n = 3). The statistical significance was determined a one-way ANOVA, followed by post-hoc Tukey's test. **** $P < 0.0001$; *ns* = not significant.

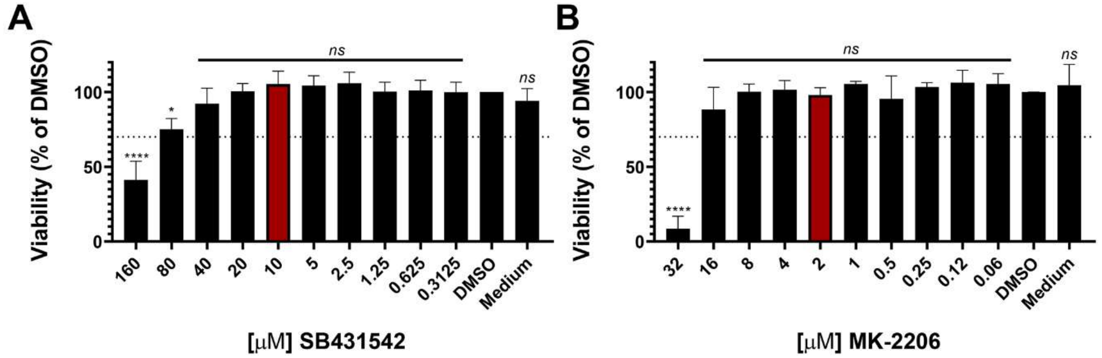

Supplementary Figure 4. Measurement of acute toxicity of TGF-β1 receptor (ALK4/5/7) and AKT inhibitors in HTR-8/SVneo cultures. Serum-starved HTR-8/SVneo cultures were treated with increasing (two-fold) concentrations of the TGF-β1 receptor (ALK4/5/7) inhibitor SB431542 **(A)**, the pan-AKT inhibitor MK-2206 **(B)** or an equivalent volume of vehicle (i.e., DMSO) for 24 h. The dashed line indicates 75% cell viability. The concentration used in subsequent experiments is identified by a red bar for each inhibitor. (A-B) Results are presented as mean [SD] calculated from three independent experiments (n = 3), each carried out in technical triplicates. The statistical significance was determined by a one-way ANOVA, followed by post-hoc Tukey's test, where **** $P < 0.0001$; *$P < 0.05$; *ns* not significant.

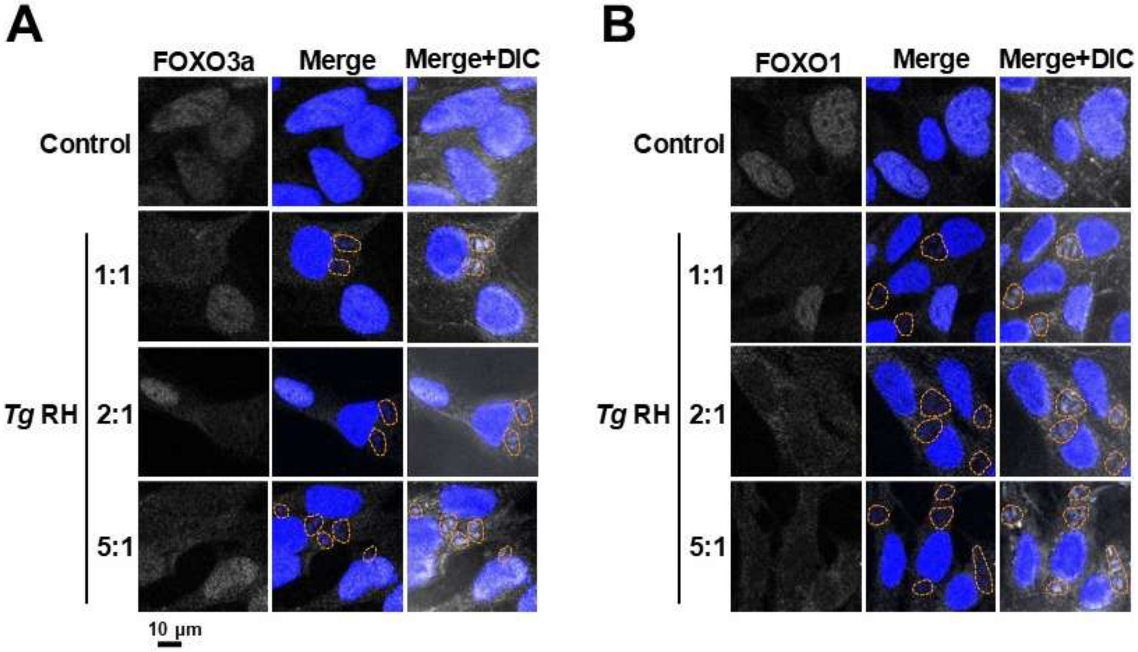

Supplementary Figure 5. ***T. gondii* triggers human EVT nuclear exclusion of FOXO3a and FOXO1 in an MOI-dependent manner**. Serum-starved HTR-8/SVneo cultures were infected with indicated MOI of *T. gondii* RH strain for 16 h. (**A-B**) Cultures were fixed and processed for confocal immunofluorescence microscopy. Samples were stained with DAPI (shown in blue), used as a nuclear marker, and for FOXO3a **(A)** or FOXO1 (**B**) (shown in white). Shown here are four times-enlarged insets. Parasitophorous vacuoles (PVs) are outlined with dashed lines to indicate the presence of parasites within infected cells. (A-B) Images are representative of three independent experiments.

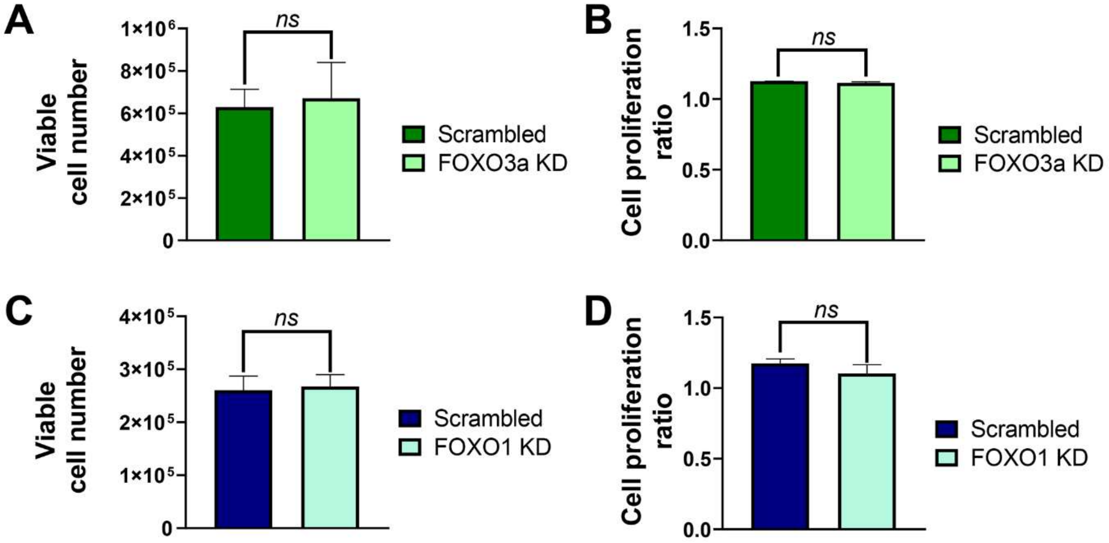

Supplementary Figure 6. Reduced expression of FOXO3a and FOXO1 does not affect viability and proliferation rates of human trophoblast cell line HTR-8/SVneo. Cell viability **(A, C)** and proliferation rates **(B, D)** were determined in serum-starved Scrambled, FOXO3a KD **(A, B)**, and FOXO1 KD **(C, D)**. (A, C) Number of live cells was quantified 16 h after invasion assays. (B, D) Cell proliferation ratio was calculated by comparing live cell counts at 0 h and 16 h post-scratch wound. (A-D) Values are presented as mean [SD] calculated from three independent experiments (n = 3). Statistical significance was determined using a two-tailed independent Student's *t*-test. *ns*, not significant.

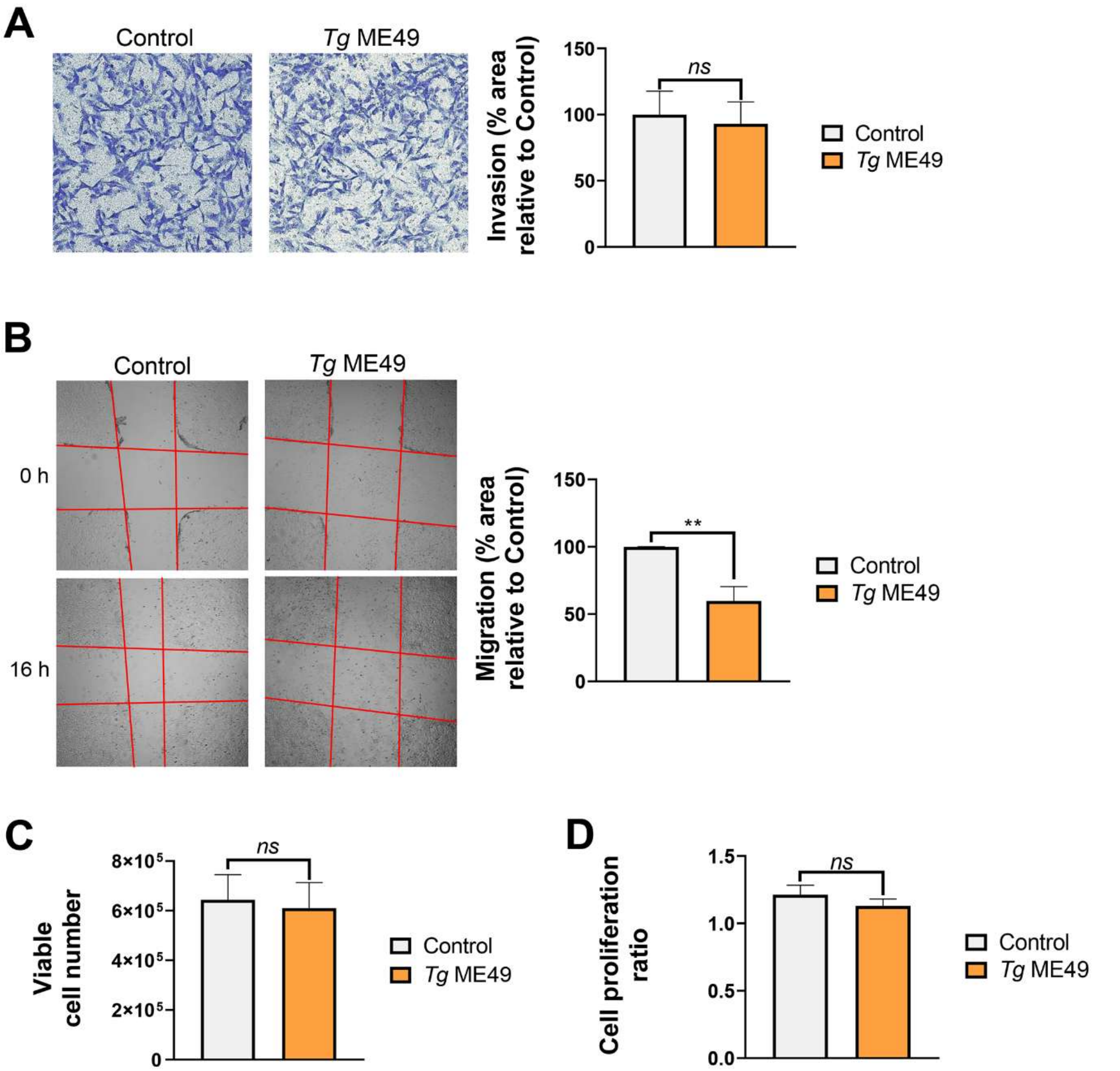

Supplementary Figure 7. Type II *T. gondii* ME49 strain downregulates migration but not invasion in the human trophoblast cell line HTR-8/SVneo. Cells were incubated in serum-free media 1 h before infection with *T. gondii* ME49 strain (MOI 5:1) or left uninfected (Control). **(A)** Collagen-based matrix invasion was monitored at 16 h.p.i. Shown here are representative images of three independent experiments (left panel) and quantification of cell invasion (right panel). **(B)**. Cell migration was assessed by wound-healing assays. Shown here are representative images of three independent experiments (left panels) and quantification of cell invasion (right panels). **(C)** Number of live cells was quantified at 16 h.p.i. (**D)** Cell proliferation ratio was calculated by comparing live cell counts at 0 h and 16 h post-scratch

wound. (A-D) Values are presented as mean [SD] calculated from three independent experiments (n = 3). Statistical significance was determined using a two-tailed independent Student's *t*-test, where $^{**}P < 0.01$; *ns* not significant.

Table S1. List of primers used for RT-qPCR experiments.

Target	Sense	Sequence (5’ 3’)
HPRT1	Forward	ATGACCAGTCAACAGGGGAC
	Reverse	TGCCTGACCAAGGAAAGCAA
MMP2	Forward	CGTCTGTCCCAGGATGACATC
	Reverse	TGTCAGGAGAGGCCCCATAG
MMP3	Forward	AGCTGAAGACTTTCCAGGGATTG
	Reverse	GTCAAACTCCAACTGTTGAAGATCC
MMP14	Forward	CGGGCTACCCCAAGTCA
	Reverse	TGATGATCACCTCCGTCTCC
TIMP2	Forward	AGTTTATCTACACGGCCCCCT
	Reverse	GGGTGATGTGCATCTTGCC
MUC1	Forward	TCTATCTCATTGCCTTGGCTGTC
	Reverse	TACTCGCTCATAGGATGGTAGG
ITGB3	Forward	TGTACCACGCGTACTGACAC
	Reverse	CTGCCACATTCACACTTGCC
CTNNB1	Forward	CCCTGCTTCAGGCGTCTGTA
	Reverse	TGCTTGCATAATGCGATTT
CDH2	Forward	CTCAGGACAAGGAAGCTGCAGAAGC
	Reverse	CAAGGCATCCTGGCCATACCA
PAPPA	Forward	AGTGGTATCCTCACCCTGCT
	Reverse	TGCAAAGGCTCGGTTGTTG
ICAM1	Forward	CTGAGGGCACCTACCTCTG
	Reverse	GGGAGAGCACATTCACGGTC
SNAIL	Forward	GACCATATGCCGCGCTCTT
	Reverse	TCGCTGTAGTTAGGCTTCCGATT
MMP9	Forward	TGGGCAGATTCCAAACCTTT
	Reverse	TCTTCCGAGTAGTTTTGGATCCA
MMP11	Forward	ACACCAATGAGATTGCACCG
	Reverse	AAGAAAAAGAGCTCGCCTCG
TUB1A	Forward	GACGACGCCTTCAACACCTTCTTT
	Reverse	AGTTGTTCGCAGCATCCTCTTTCC

Table S2. List of shRNA clones used for reverse-genetics experiments.

**shRNA clones**		
**Target**	**Catalog # Genecopeia**	**Description**
Scrambled (Scr.)	CS-HCTR001-LVRU6MP	shRNA scrambled control clone for psi-LVRU6MP
FOXO3	CS-HSH005759-LVRU6MP-02	shRNA clone set of 3 constructs against 3 variants for human FOXO3 (ENST00000540898.1, NM_001455.4, and NM_201559.3) in lentiviral psi-LVRU6MP vector with U6 promoter, mCherry, puromycin
FOXO1	CS-HSH127365-LVRU6GP-01	shRNA clone set of 3 constructs against custom variants for human FOXO1 (NM_002015.4, ENST00000473775.1, ENST00000655267.1, & ENST00000660760.1) in lentiviral psi-LVRU6GP vector with U6 promoter, eGFP, puromycin
